# Epigenomics Nutritional Insights of *Crocus sativus* L.: Computational Analysis of Bioactive Molecules Targeting DNA Methyltransferases and Histone Deacetylases

**DOI:** 10.3390/ijms26157575

**Published:** 2025-08-05

**Authors:** Alessia Piergentili, Paolo Roberto Saraceni, Olivia Costantina Demurtas, Barbara Benassi, Caterina Arcangeli

**Affiliations:** 1 Institute for Neuroscience and Medicine (INM-9), Forschungszentrum Jülich, Wilhelm-Johnen-Straße, 52428 Jülich, Germany; a.piergentili@fz-juelich.de; 2 Department of Neurology, University Hospital Aachen, RWTH Aachen, Pauwelsstraße 30, 52074 Aachen, Germany; 3 Department for Sustainability, Italian National Agency for New Technologies, Energy and Sustainable Economic Development (ENEA) Casaccia Research Center, Via Anguillarese 301, 00123 Rome, Italy; paolo.saraceni@enea.it (P.R.S.); olivia.demurtas@enea.it (O.C.D.)

**Keywords:** saffron, DNMTs, HDAC2, Sirtuin-1, molecular docking, molecular dynamics, crocin, crocetin, picrocrocin, safranal

## Abstract

Saffron (*Crocus sativus* L.) contains bioactive compounds with potential health benefits, including modulation of protein function and gene expression. However, their ability to tune the epigenetic machine remains poorly understood. This study employs molecular docking (AutoDock Vina 1.4), dynamics simulations, and MM/PBSA calculations to investigate the interactions between four saffron-derived molecules—crocetin, beta-D-glucosyl trans-crocetin, picrocrocin and safranal—and four epigenetic enzymes—DNMT1, DNMT3a, HDAC2, and SIRT1. Our in silico screening identifies beta-D-glucosyl trans-crocetin, one of the saffron’s crocins, as a potential DNMT1 inhibitor. Along with crocetin, it also shows the ability to inhibit HDAC2 and activate SIRT1. Picrocrocin displays a resveratrol-like ability to activate SIRT1. None of the saffron-derived compounds effectively bind or inhibit DNMT3a. Among the tested molecules, safranal shows no interaction with the selected epigenetic targets. These findings highlight saffron’s nutriepigenomic potential and emphasize the need for functional validation within relevant in vitro and in vivo experimental methodologies.

## 1. Introduction

Diet and food-derived biomolecules can directly affect cell physiology and help preventing chronic diseases at multiple levels, by tuning gene expression and by regulating the epigenetic machinery, as experimentally substantiated by nutrigenomic and nutriepigenomic studies [1,2,3]. The class of polyphenols (green tea-derived catechins, quercetin, myricetin), soy isoflavones (genistein), parthenolide, curcumin, resveratrol, isothiocyantes, and butyrate are the phytochemicals associated with the greatest nutrition-derived health benefits, with a demonstrated potential to elicit specific epigenetic response [4,5,6].

Recently, different experimental and human studies have proved the capacity of saffron, a spice derived from the flowers of *Crocus sativus* L.—a sterile triploid plant traditionally cultivated from Spain to Kashmir [7]—to ameliorate conditions and symptoms related to diabetes, cardiovascular disease, depression, and neurodegenerative conditions [8,9], and also to act as promising anti-neoplastic agents [10,11,12]. The interest in saffron-induced health benefits has led to various applications, either through the characterization of its phytochemicals as novel therapeutic agents or through its use as a functional food [13,14]. The latter approach is supported by saffron’s potential nutrigenomic and nutriepigenomic properties [15,16], which may contribute to the prevention of chronic diseases and the delay of aging.

Its bioactive phytochemicals, mainly crocins (a group of glycosylated pigments), picrocrocin, crocetin (crocins aglycone), and safranal, belonging to the class of apocarotenoids, have been studied and hypothesized to promote the different health protective functions through their ability to act on specific molecular pathways at both protein and gene levels [17].

Preliminary evidence also suggests that saffron biomolecules may exert effects at the epigenetic level [18]; however, current experimental data remain limited and insufficient to conclusively demonstrate a strong direct nutriepigenomic role that would substantiate saffron’s potential as a functional food. Epigenetics plays a major role in connecting human DNA with external stimuli, including food and nutrients, thus representing a valuable target mechanism to be explored to gain insights into the health effects promoted by saffron.

Epigenetics encompasses different levels of regulation, including chromatin remodeling by histone post-translational modifications (PTMs), DNA methylation, and non-coding RNA-mediated gene regulation [19].

Following specific PTMs, histones change the architecture of nucleosomes and regulate genome plasticity in response to external stimuli, including nutrients and food-derived biomolecules [20]. By chromatin structure modification, gene expression can be tuned, according to the cell response to be activated. Post-translational modifications entail multiple mechanism (s), including histone acetylation (HA) [21]; it involves the addition of an acetyl group to the ε-amino group of lysine residue in the protruding histone tails by histone acetyl transferases (HATs) and leads to transcriptional activation [22,23]. By contrast, histone deacetylases (HDACs) trigger the acetyl removal from lysine residue and drive transcriptional repression [24,25]. In humans, different deacetylating enzymes have been identified [24,26]: class I (HDAC 1-3 and 8); class II (HDAC 4-, 7, 9,10); class III (sirtuins, SIRT 1-7), and class IV (HDAC 11). SIRT1 also deacetylates specific transcription factors and enzymes, such as the nuclear factor kappa-light-chain-enhancer of activated B cells (NF-Kb) and tumor suppressor protein p53, to influence their subcellular localization and activity [27,28], thus amplifying the panel of molecular pathways set under its epigenetic control. Sirtuins are regulated by physical exercise and diet [29,30]; the caloric restriction (CR) represents the most studied dietary intervention linked to SIRT1 upregulation [31,32], thus directly connecting this HDAC class to the extension of life span and anti-aging function. Moreover, specific dietary compounds, mainly resveratrol and polyphenols, directly interact and promote SIRT1 activity [33,34]. Diet and food bioactives also target class I deacetylases [35]. In this context, HDAC2 has been proved to be specifically inhibited by a wide range of food-derived biomolecules, such as kaempferol—a flavonol abundantly found in tea, broccoli, apples, strawberries, and beans—rosmarinic acid, and oleuropein from virgin olive oil [36,37,38]. Also, CR affects HDAC2 functionality, as highlighted in murine neuronal tissue where CR prevented the age-related increase in HDAC2 levels in the hippocampus from C57Bl6J male mice [39].

Once chromatin has been rendered accessible by histone PTMs, it becomes permissive to the transcriptional machinery, thereby allowing the regulation of gene expression through DNA methylation. DNA methylation predominantly occurs at CpG islands within promoter regions and is mediated by DNA methyltransferases (DNMTs), including DNMT1, DNMT3a, and DNMT3b. These enzymes are responsible for the establishment and maintenance of DNA methylation patterns, primarily leading to transcriptional repression of genes associated with hypermethylated promoters [40]. Several dietary components may directly modulate DNMT expression and activity, while dietary methyl donors can indirectly influence DNMT enzymatic ability by altering intracellular levels of S-adenosylmethionine, the primary methyl group donor for DNA methylation reactions [41,42,43]. These findings strengthen the key role of DNMTs in promoting health protection functions by diet and nutritional supplementation.

The present in silico study integrates molecular docking and dynamics simulations to characterize the interaction between crocetin and its mono-glycosylated derivative beta-D-glucosyl trans-crocetin (one of the crocins of *Crocus sativus*), picrocrocin and safranal, and key human epigenetic modulators: DNMT1, DNMT3a, HDAC2, and SIRT1 enzymes. The saffron-derived molecules were chosen according to their abundance in stigmas and to their known bioavailability and potential re-glycosylation in human cells [44,45]. The final goal is to provide computational proof-of-concept for the nutriepigenomic property of these compounds, suggesting their possible role in mediating the epigenetic effects of a saffron-enriched diet or a saffron-based nutraceutical formulation in anti-aging and disease prevention contexts.

## 2. Results

### 2.1. Evaluation of Saffron Compounds as Epigenetic Enzyme Ligands Through Docking

The saffron apocarotenoids—beta-D-glucosyl trans-crocetin, crocetin, picrocrocin, and safranal (Figure S1)—were evaluated for their potential binding affinity with two DNA methyltransferases (DNMT1 and DNMT3a) and two deacetylases enzymes (HDAC2 and SIRT1) by molecular docking simulations. To evaluate the binding affinity of these bioactive molecules, their predictive binding affinity (docking score) was compared with that of reference compounds: (i) sinefungin (SFG), a natural inhibitor of the DNMTs, (ii) the 20Y ligand, an inhibitor of HDAC2 and (iii) the resveratrol (STL) molecule, an activator of SIRT1. As shown in Table 1, among the tested biomolecules, safranal exhibits very low affinity, with docking score values higher than −6.0 kcal/mol, for all the epigenetic enzymes considered in this study. As expected, the natural ligands (SFG, 20Y, and STL) show high affinity (Table 1) for their respective targets in agreement with the literature data [46,47,48]: DNMT1 (docking score = −9.3 kcal/mol), DNMT3a (docking score = −8.1 kcal/mol), HDAC2 (docking score = −8.4 kcal/mol), and SIRT1 (docking score = −8.0 kcal/mol). Crocetin and beta-D-glucosyl trans-crocetin (named crocin for simplicity) display docking scores indicative of potential interaction with both DNMTs and HDAC2, as well as with SIRT1, showing binding affinity values comparable or higher than those of the reference compounds. The docking score values calculated for the SIRT1 in a complex with resveratrol, crocetin, and safranal are in agreement with values previously reported in the literature [47,48]. Picrocrocin shows a lower predicted binding affinity for DNMT3a and SIRT1, and a limited interaction with HDAC2.

### 2.2. Structural Stability of Ligand–Epigenetic Target Complexes via Molecular Dynamics Simulations

Ligand–receptor binding is a dynamic process, whereas in docking simulations, the receptor is treated as rigid, leaving the precise docking conformation uncertain. To address this limitation, we employed molecular dynamics (MD) simulations, which enable a more extensive exploration of the conformational landscape and provide a deeper evaluation of the complex’s stability. Each docked complex was subjected to 200 ns of MD simulation in an explicit solvent under physiological cellular conditions (300 K temperature, 150 mM NaCl, neutral nucleic pH) to assess the overall stability of the system.

#### 2.2.1. Quality Assessment of the MD Simulations

To assess the quality of the simulation, the root mean square deviation (RMSD) values of the alpha carbon (Cα) atoms of the epigenetic target were calculated during the simulation run. As shown in Figure S2, after about 100 ns of the simulation, all the RMSD curves reach a plateau, indicating that all the epigenetic targets have achieved structural and conformational stability. This behavior indicates that a 200 ns simulation is sufficient for the epigenetic targets to achieve conformational stability and is appropriate for evaluating the stability of the docking poses. Notably, while the RMSD profile of SIRT1 in a complex with safranal (SFR) also stabilizes after 100 ns, it displays relatively high RMSD values, suggesting significant conformational rearrangements and dynamic fluctuations. The root mean square fluctuation (RMSF) analysis, which measures the average positional deviations of individual residues over time and identifies flexible regions, further supports these observations. As shown in Figure S3 in most complexes, including the control SIRT1:STL, fluctuations remain low and localized. In contrast, the SIRT1:SFR complex shows markedly increased flexibility across nearly the entire protein sequence. This effect is especially pronounced in the N-terminal domain (NTD, residues 174–240), particularly within the known allosteric region (residues 195–240) [49], where elevated RMSF values suggest a destabilizing influence exerted by safranal binding, likely due to the disruption of allosteric communication pathways. Additionally, increased flexibility within the catalytic domain (CD, residues 241–500) further indicates a ligand-induced global conformational impact.

Overall, the RMSD and RMSF analyses demonstrate that, despite localized fluctuations observed in some complexes, particularly SIRT1:SFR, the MD simulations achieve sufficient structural stability to allow for the reliable analysis of ligand-binding poses and their stability.

#### 2.2.2. Stability of Ligand-Binding Poses

**Figure 1:**
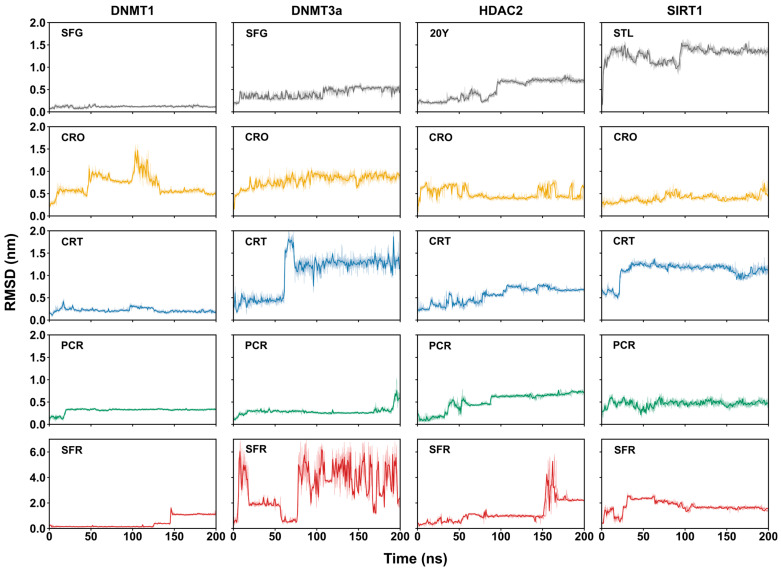
The RMSD values of the ligand atoms in a complex with the epigenetic targets as a function of simulation time. The shaded areas indicate the range of variation in the RMSD curves.

The variation in the ligand poses with respect to the epigenetic enzymes was assessed by calculating the RMSD’s values of ligand heavy atoms (Figure 1), the minimum distance profiles (Figure S4), and the average number of contacts within 0.4 nm between ligands and binding site residues (Table 2). Distances shorter than 0.4 nm indicate close-range interactions, typically associated with non-covalent contacts such as hydrogen bonds, salt bridges, or van der Waals interactions. Therefore, a higher number of contacts within this distance range suggests a stronger and more stable ligand–receptor complex formation.

The control complexes DNMT1:SFG and DNMT3a:SFG display stable RMSD profiles (Figure 1) and a high number of close contacts (463.60 ± 25.82 and 367.27 ± 31.44, respectively), with consistent minimum distances (~0.16 nm) (Figure S4), indicating that, as expected, the SFG ligand maintains a consistent binding pose throughout the simulation. HDAC2:20Y shows pose stabilization after ~100 ns, reaching 149.04 ± 20.29 contacts and a ~0.25 nm average distance (Figure S4). In contrast, the SIRT1:STL complex undergoes significant conformational rearrangement (ligand RMSD >1.5 nm) and forms few stable contacts (74.56 ± 22.52). This finding is consistent with the known requirement of two resveratrol molecules for proper stabilization, which interact with multiple sites in NTD of SIRT1 [49,50]. Still, the single resveratrol molecule remains relatively close to the binding pocket (~0.25 nm).

Among saffron-derived compounds, crocin shows stable binding with DNMT1 and HDAC2, while crocetin displays stable poses with DNMT3a, HDAC2, and SIRT1. Picrocrocin remains stable across all targets, although in DNMT3a, the ligand gradually dissociates after 100 ns, with distances exceeding 0.5 nm. Safranal fails to establish a stable pose in any complex, as indicated by high RMSD fluctuations and unstable distance profiles (Figure 1 and Figure S4); notably, the DNMT1:SFR complex destabilizes after ~140 ns. Contact analysis shown in Table 2 supports these findings. By the comparison with their respective controls, crocin forms numerous contacts with DNMT1 (371.31 ± 30.59), HDAC2 (241.10 ± 24.48), and SIRT1 (202.24 ± 30.84). Crocetin and picrocrocin show several interactions with HDAC2 (192 ± 19.97) and SIRT1 (144 ± 27.16), whereas safranal shows minimal interaction with all the epigenetic targets. Notably, the higher number of contacts observed for crocin, crocetin, and picrocrocin in a complex with SIRT1, compared to STL, suggests the occurrence of alternative or stronger binding modes.

Taken together, these analyses indicate a variable binding behavior of the saffron-derived compounds across different epigenetic targets. Crocin, crocetin, and picrocrocin demonstrate the ability to form stable and potentially strong complexes with specific enzymes, supporting their potential as multitarget epigenetic modulators. In contrast, safranal appears unable to retain a stable binding pose in any complex, suggesting a limited role in modulating epigenetic enzyme activity.

#### 2.2.3. Binding Mode Dynamics and Conformational Variability

**Figure 2:**
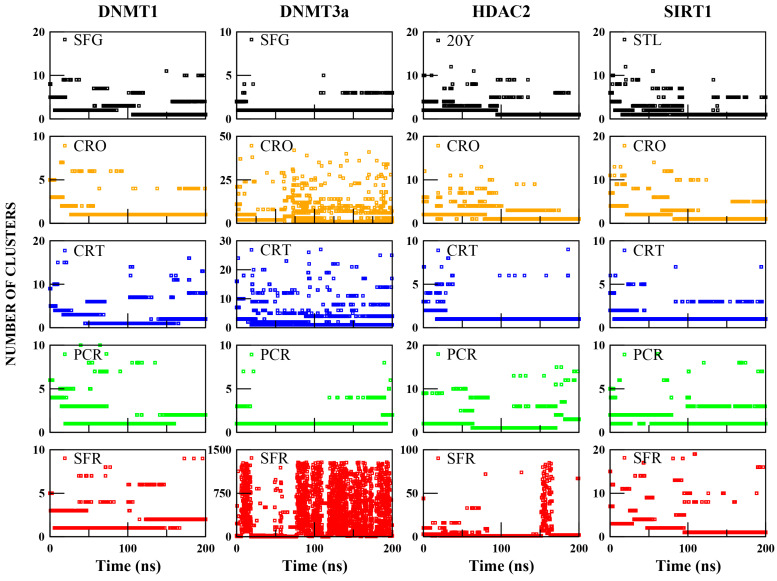
The number of clusters as a function of simulation time and their occupancy throughout the trajectory of the ligand–target complexes.

**Figure 3:**
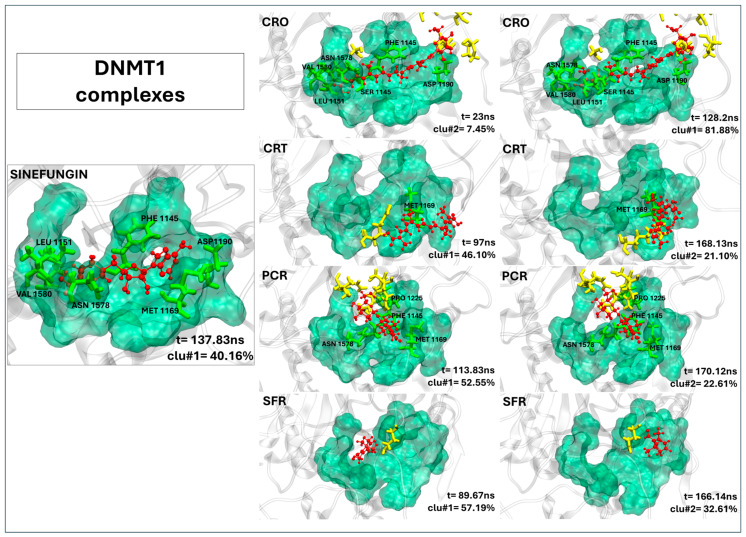
The representative structures of MD trajectory extracted from the two most populated clusters of DNMT1:CRO, DNMT1:CRT, DNMT1:PCR, and DNMT1:SFR. For comparison, the representative structure taken from the first most populated cluster of DNMT1:SFG control is also shown. Binding pocket residues are colored in green, extra binding pocket residues in yellow, and ligands in red. A close-up view of the ligand–target binding interface is provided in Figure S7A, highlighting key interactions and atomic distances for the DNMT1:CRO complex.

**Figure 4:**
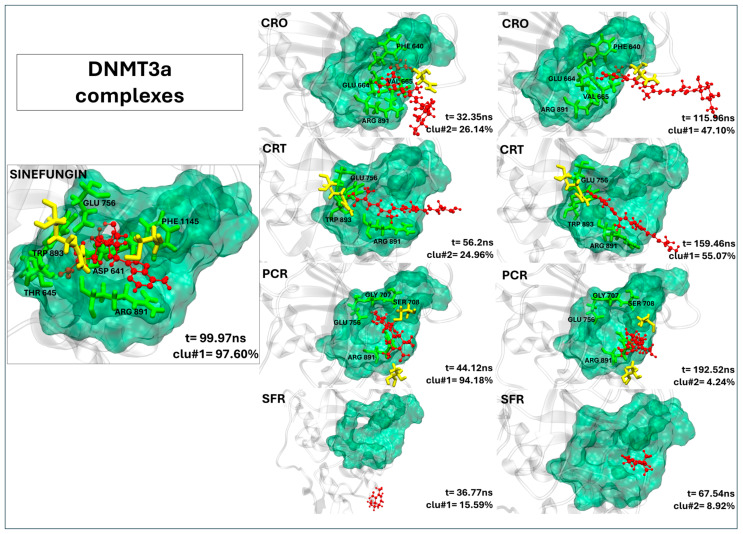
The representative structures of MD trajectory extracted from the two most populated clusters of DNMT3a:CRO, DNMT3a:CRT, DNMT3a:PCR, and DNMT3a:SFR. For comparison, the representative structure taken from the first most populated cluster of DNMT3a:SFG control is also shown. Binding pocket residues are colored in green, extra binding pocket residues in yellow, and ligands in red.

**Figure 5:**
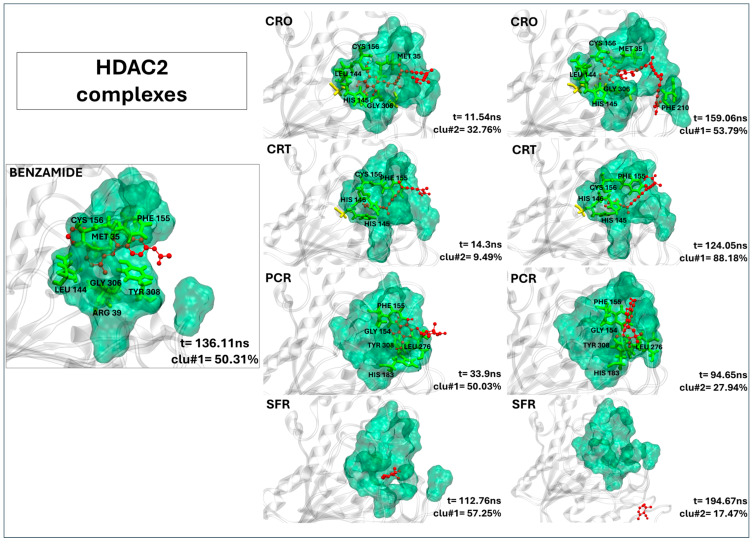
The representative structures of MD trajectory extracted from the two most populated clusters of HDAC2:CRO, HDAC2:CRT, HDAC2:PCR, and HDAC2:SFR. For comparison, the representative structure taken from the first most populated cluster of HDAC2:20Y control is also shown. Binding pocket residues are colored in green, extra binding pocket residues in yellow, and ligands in red. A close-up view of the ligand–target binding interface is provided in Figure S7B,C, highlighting key interactions and atomic distances for the HDAC2:CRO and HDAC2:CRT complexes.

**Figure 6:**
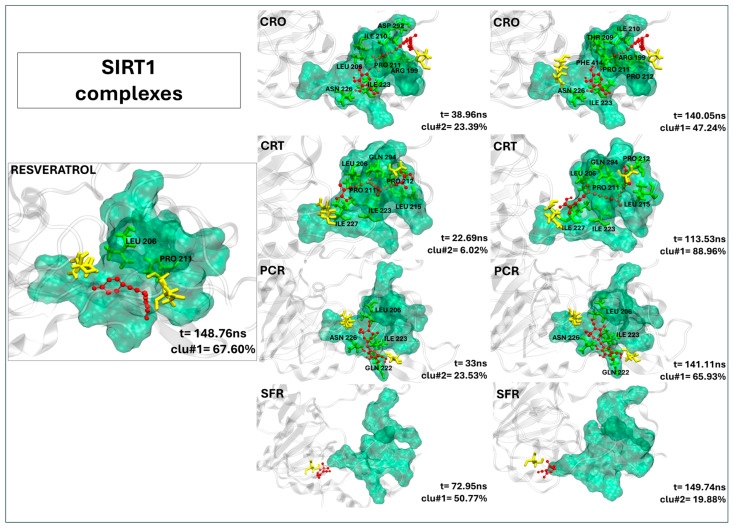
The representative structures of MD trajectory extracted from the two most populated clusters of SIRT1:CRO, SIRT1:CRT, SIRT1:PCR, and SIRT1:SFR. For comparison, the representative structure taken from the first most populated cluster of SIRT1:SFG control is also shown. Binding pocket residues are colored in green, extra binding pocket residues in yellow, and ligands in red. A close-up view of the ligand–target binding interface is provided in Figure S7D–F, highlighting key interactions and atomic distances for the SIRT1:CRO, SIRT1:CRT, and SIRT1:PCR complexes.

To address the limitation of the RMSD analysis, which, while providing a useful estimate of pose stability over time, does not distinguish between distinct binding conformations, a clustering analysis was performed. By grouping similar conformations along the trajectory, this approach allows the identification of the dominant binding modes and the structural variability within the ligand–receptor complexes. The distribution (Figure 2) and size (Table S1) of the clusters reflect the degree of stability or conformational changes in ligand binding, helping to distinguish between well-defined interactions and highly dynamic or unstable poses. In addition, representative structures (Figures 3–6) extracted from the most populated clusters are shown to visually inspect the main conformations adopted by the ligands, along with the specific contacts formed with receptor residues and their residence time within the binding pocket (Tables 3–6). These data, analyzed in direct comparison with the control complexes, provide a more refined understanding of binding mode stability and interaction specificity.

As shown in Figure 2, the control complexes (DNMT1:SFG, DNMT3a:SFG, HDAC2:20Y, SIRT1:STL) show low cluster variability over time. DNMT3a:SFG mainly adopts one cluster (97% of the simulation), while the others adopt two dominant clusters covering 71% (DNMT1:SFG), 87% (HDAC2:20Y), and 85% (SIRT1:STL) of the trajectories (Table S1). Representative structures are shown in Figure S6, confirming that all control ligands maintain stable binding poses, with only minor orientation changes within the pocket.

In contrast, saffron compounds show more variable clustering. Except for DNMT3a:SFR, over 80% of structures fall within the first three clusters (Table S1). However, complexes like DNMT3a:CRO, DNMT3a:CRT, DNMT3a:SFR, and HDAC2:SFR form many clusters, suggesting low structural stability and high pose variability (Figure 2, Table S1). Representative structures for DNMT1, DNMT3a, HDAC2, and SIRT1 bound to the saffron compounds are shown in Figure 3, Figure 4, Figure 5, and Figure 6, respectively.

#### 2.2.4. Crocin as a Promising DNMT1 Inhibitor Candidate

As clearly shown in Figure 3, among the tested molecules, only crocin demonstrates a binding conformation within DNMT1 that closely mimics the binding pose of the reference inhibitor sinefungin. The crocin is well accommodated in the catalytic pocket and establishes a stable interaction network with several key active site residues, including PHE1445, SER1146, GLY1147, GLY1150, LEU1151, GLU1168, MET1169, ASP1190, LEU1247, ASN1578, ALA1579, and VAL1580 (see Table 3). Notably, its prolonged residence time, comparable to that of the reference ligand, further supports its stable binding mode (see Table 3). Although picrocrocin appears to occupy the binding site (Figure 3), it predominantly interacts with residues located outside the canonical pocket, as detailed in Table 3. The crocetin and safranal ligands fail to properly accommodate within the binding site, highlighting poor structural compatibility and suggesting a lack of inhibitory activity.

**Table 3:** The contact frequency analysis of ligands in a complex with DNMT1 and their residence time within the binding pocket. Bold text indicates residues belonging to the active site (binding pocket). The frequency of contacts (distance < 0.4 nm) between each target residue and the ligands is expressed as the percentage of simulation time during which the contact persists (only values >80% are reported).

DNMT1’s Residues	SFG	CRO	CRT	PCR	SFR
PRO 615	-	99.12	-	-	-
LYS 617	-	88.53	-	-	-
ALA 618	-	85.44	-	-	-
**PHE 1145**	**100.00**	**99.92**	-	**89.68**	-
**SER 1146**	**99.93**	**100.00**	-	-	-
**GLY 1147**	**98.21**	**87.24**	-	-	-
**CYS 1148**	**95.95**	-	-	-	-
**GLY 1149**	**97.45**	-	-	-	-
**GLY 1150**	**99.97**	**99.41**	-	-	-
**LEU 1151**	**95.81**	**97.69**	-	-	-
**GLU 1168**	**100.00**	**94.53**	-	-	-
**MET 1169**	**99.93**	**85.07**	**91.37**	**82.70**	-
**TRP 1170**	**89.29**	-	-	-	-
**GLU 1189**	**76.98**	-	-	-	-
**ASP 1190**	**100.00**	**91.84**	-	-	-
**CYS 1191**	**100.00**	-	-	-	-
ASN 1192	-	97.86	-	-	-
GLY 1223	-	86.94	-	98.61	-
PRO 1224	-	-	-	82.80	-
**PRO 1225**	-	-	-	**98.96**	-
CYS 1226	-	-	-	96.62	-
GLN 1227	-	-	-	90.51	91.26
ASN 1245	-	84.84	-	-	-
**LEU 1247**	**97.45**	-	-	-	-
ASN 1267	-	-	-	98.45	-
VAL 1268	-	-	-	90.33	-
ARG 1574	-	-	82.09	-	-
**ASN 1578**	**99.85**	**96.32**	-	**99.92**	-
**ALA 1579**	**96.90**	**99.59**	-	-	-
**VAL 1580**	**99.50**	**99.93**	-	-	-
**Contacts ^(a)^**	17 (17)	18 (12)	3 (1)	11 (4)	1 (0)
**Residence time ^(b)^**	200 ns	199.93 ns	0.00 ns	0.00 ns	0.00 ns

^(a)^ Total number of persistent contacts per ligand, with those involving active site residues indicated in parentheses. ^(b)^ Ligand residence time (in nanosecond, ns) within the binding pocket.

#### 2.2.5. Lack of Effective Binding of Saffron Compounds to DNMT3a

In DNMT3a, none of the saffron-derived compounds successfully replicate the binding mode of sinefungin, as shown in Figure 4. Although crocin and crocetin localize near the binding pocket, they establish few meaningful contacts with key catalytic residues (Table 4). Only sporadic contacts are registered, with crocin forming five persistent interactions (four involving active site residues) and crocetin forming four contacts (three at the active site). Picrocrocin fails to insert into the active site, and safranal remains distant from any relevant interaction region. As clearly shown in Table 4, none of the ligands exhibited a residence time within the binding pocket comparable to that of the control ligand, with values being either zero or covering at most 20% of the simulation time. Given the absence of stable, high-occupancy interactions with catalytic residues, these compounds are unlikely to exert a relevant inhibitory effect on DNMT3a.

**Table 4:** The contact frequency analysis of ligands in a complex with DNMT3a and their residence time within the binding pocket. Bold text indicates residues belonging to the active site (binding pocket). The frequency of contacts (distance < 0.4 nm) between each target residue and the ligands is expressed as the percentage of simulation time during which the contact persists (only values >80% are reported).

DNMT3a’s Residues	SFG	CRO	CRT	PCR	SFR
**PHE 640**	**99.65**	**95.12**	-	-	-
**ASP 641**	**100.00**	-	-	-	-
**ILE 643**	**98.59**	-	-	-	-
**ALA 644**	**90.81**	-	-	-	-
**THR 645**	**100.00**	-	-	-	-
**GLU 664**	-	**99.83**	-	-	-
**VAL 665**	-	**91.72**	-	-	-
**GLY 707**	**99.14**	-	-	**91.01**	-
**SER 708**	**99.98**	-	-	**94.95**	-
**PRO 709**	**83.04**	-	-	-	-
CYS 710	91.56	-	-	83.14	-
ASN 711	-	83.85	-	-	-
**GLU 756**	**100.00**	-	**98.18**	**84.22**	-
ASN 757	90.89	-	-		-
ARG 792	91.22	-	97.95	-	-
THR 834	-	-	-	86.99	-
**ARG 891**	**99.53**	**97.13**	**98.34**	**91.44**	-
**SER 892**	**99.25**	-	-	-	-
**TRP 893**	**100.00**	-	**83.23**	-	-
**Contacts ^(a)^**	15 (12)	5 (4)	4 (3)	6 (4)	0 (0)
**Residence time ^(b)^**	194.31 ns	0.00 ns	40.42 ns	0.00 ns	0.00 ns

^(a)^ Total number of persistent contacts per ligand, with those involving active site residues indicated in parentheses. ^(b)^ Ligand residence time (in nanosecond, ns) within the binding pocket.

#### 2.2.6. Crocin and Crocetin Exhibit Stable and Persistent Binding Within the HDAC2 Active Site

As shown in Figure 5, both crocin and crocetin form stable complexes with HDAC2, adopting binding conformations closely resembling those of the reference inhibitor (20Y). Specifically, crocin establishes multiple stable contacts with residues in the binding pocket, such as PHE155, LEU144, HIS145, GLY306, and PHE210 (Table 5). Crocetin also fits well within the active site, forming 11 close contacts, including interactions with PHE210 (Table 5), a residue consistently involved in ligand binding and crucial for the stability of the complex. Notably, residue PHE210 remains consistently involved throughout the simulation in both crocin and crocetin complexes, contributing significantly to interaction stability. Remarkably, the residence times of crocin and crocetin suggest that both ligands persistently occupied the binding pocket throughout the full simulation time (see Table 5). By contrast, picrocrocin and safranal interact only weakly with HDAC2. While picrocrocin forms five contacts (all involving active site residues), its binding is shallow and structurally unstable. Safranal, on the other hand, fails to engage in any persistent interaction, suggesting negligible affinity for the HDAC2 binding site.

**Table 5:** The contact frequency analysis of ligands in a complex with HDAC2 and their residence time within the binding pocket. Bold text indicates residues belonging to the active site (binding pocket). The frequency of contacts (distance < 0.4 nm) between each target residue and the ligands is expressed as the percentage of simulation time during which the contact persists (only values >80% are reported).

HDAC2’s Residues	20Y	CRO	CRT	PCR	SFR
**MET 35**	**98.74**	**97.98**	**95.66**	-	-
ARG 39	89.98	-	-	-	-
**ASP 104**	-	-	**91.59**	-	-
GLY 143	-	90.34	92.04	-	-
**LEU 144**	**90.78**	**97.46**	-	-	-
**HIS 145**	-	**85.45**	**98.14**	-	-
**HIS 146**	-	-	**97.35**	-	-
**GLY 154**	-	**97.53**	**99.69**	**93.04**	-
**PHE 155**	**98.64**	**99.46**	**99.95**	**96.65**	-
**CYS 156**	**93.29**	**95.75**	**92.62**	-	-
**HIS 183**	-	-	-	**98.28**	-
**PHE 210**	-	**88.18**	**99.95**	-	-
PRO 211	-	-	89.85	-	-
**LEU 276**	-	-	-	**94.36**	-
GLY 305	-	96.63	-	-	-
**GLY 306**	**95.80**	**99.67**	**96.98**	-	-
GLY 307	-	88.33	-	-	-
**TYR 308**	**95.17**	-	-	**88.64**	-
**Contacts ^(a)^**	7 (6)	11 (8)	11 (9)	5 (5)	0 (0)
**Residence time ^(b)^**	191.61 ns	200.00 ns	200.00 ns	79.62 ns	2.80 ns

^(a)^ Total number of persistent contacts per ligand, with those involving active site residues indicated in parentheses. ^(b)^ Ligand residence time (in nanosecond, ns) within the binding pocket.

#### 2.2.7. Crocin, Crocetin, and Picrocrocin Are Promising Candidates as SIRT1 Activators

The visual inspection of the MD trajectories (Figure 6) reveals that crocin and crocetin exhibit high binding stability within the SIRT1 allosteric activation region. Both crocin and crocetin surpass the control resveratrol in terms of contact persistence and binding site coverage (Table 6). Crocin forms 15 persistent contacts with the receptor, 11 of which involve residues in the allosteric sites (ASN226, ASP292, and ASP298). Key interactions include ILE210 (99.92%), GLN294 (99.97%), and PRO211 (97.42%), indicating strong and stable accommodation at the domain interface. Crocetin also maintains a favorable binding pose, forming 11 stable contacts, 9 of which involve residues of the active site. Prominent interactions include ILE223, ILE227, and PRO212, highlighting crocetin’s potential to stabilize SIRT1’s active conformation similarly to crocin. Even if picrocrocin forms only six persistent contacts, four of which involving the active site including two allosteric residues (GLN222, 96.48% and ASN226, 98.82%), the binding pose resembles that observed by the control ligand, as shown in Figure 6, suggesting a resveratrol-like binding. On the contrary, safranal shows a single contact (with SER454), and none within the functional activation sites. The ligand residence times, reported in Table 6, indicate that both crocin and crocetin remain bound within the SIRT1-binding pocket throughout the entire 200 ns simulation, while picrocrocin remains within the active site for over 60% of the trajectory. These findings suggest that crocin, crocetin, and picrocrocin can consistently stabilize the closed conformation of SIRT1 necessary for activation, supporting their role as promising activator candidates.

**Table 6:** The contact frequency analysis of ligands in a complex with SIRT1 and their residence time within the binding pocket. Bold text indicates residues belonging to the active site (binding pocket). The frequency of contacts (distance < 0.4 nm) between each target residue and the ligands is expressed as the percentage of simulation time during which the contact persists (only values >70% are reported). The SIRT1-binding pocket comprises multiple sites within the N-terminal domain (NTD), known as site 1 (S1), site 2 (S2), and site 3 (S3), which contribute to its allosteric activation. These sub-sites are indicated in the table using superscript labels.

SIRT1’s Residues	STL	CRO	CRT	PCR	SFR
ARG 199	-	97.32	-	-	-
**LEU 206**	**71.23**	**70.98**	**87.78**	**88.86**	-
**THR 209**	-	**85.59**	**71.47**	-	-
**ILE 210**	-	**99.92**	-	-	-
**PRO 211**	**73.39**	**97.42**	**84.83**	-	-
**PRO 212**	-	**94.17**	**92.52**	-	-
PRO 213	-	96.27		-	-
**LEU 215**	-	-	**79.18**	-	-
THR 219	-	-	-	91.28	-
**GLN 222 ^(S2)^**	-	-	-	**96.48**	-
**ILE 223**	-	**80.40**	**91.75**	**99.74**	-
**ASN 226 ^(S2)^**	-	**77.78**	**78.98**	**98.82**	-
**ILE 227**	-	**71.11**	**92.59**	-	-
**ASP 292 ^(S3)^**	-	**86.70**	-	-	-
**GLN 294**	-	**99.97**	**74.39**	-	-
ALA 295	-	-	73.05	-	-
**ASP 298 ^(S3)^**	-	**71.83**	-	-	-
**PHE 414**	-	**90.03**	-	-	-
GLY 415	86.74	-	-	-	-
GLU 416	71.04	-	-	-	-
ARG 446	92.73	72.13	95.06	89.61	-
SER 454	-	-	-	-	71.03
**Contacts ^(a)^**	5 (2)	15 (11)	11 (9)	6(4)	1 (0)
**Residence time ^(b)^**	192.64 ns	199.06 ns	200.00 ns	125.02 ns	3.10 ns

^(a)^ Total number of persistent contacts per ligand, with those involving active site residues indicated in parentheses. ^(b)^ Ligand residence time (in nanosecond, ns) within the binding pocket.

### 2.3. Binding Affinity Quantified by MM/PBSA Analysis

The potential binding affinity of saffron-derived ligands to the epigenetic targets was assessed by calculating the binding free energies using the MM/PBSA (Molecular Mechanics/Poisson–Boltzmann Surface Area) method from the entire simulation (0–200 ns). The results are summarized in Table 7 and Figure S8.

Crocin demonstrates high binding affinity to DNMT1 (−42.39 kcal/mol), HDAC2 (−45.00 kcal/mol), and SIRT1 (−50.17 kcal/mol), suggesting its potential to modulate at least three targets simultaneously. Similarly, crocetin shows a broad interaction profile, with strong binding affinities for HDAC2 (−47.23 kcal/mol) and for SIRT1 (−34.49 kcal/mol). Picrocrocin demonstrated a binding free energy of −23.31 kcal/mol for SIRT1, which is comparable to the binding free energy of the reference compound (−25.80 kcal/mol). The binding free energy values obtained for safranal clearly indicate its very low affinity toward all the tested epigenetic targets. This finding may be related to the intrinsic volatility of safranal and its limited ability to form stable interactions, as previously highlighted in analytical studies [51].

**Figure 7:**
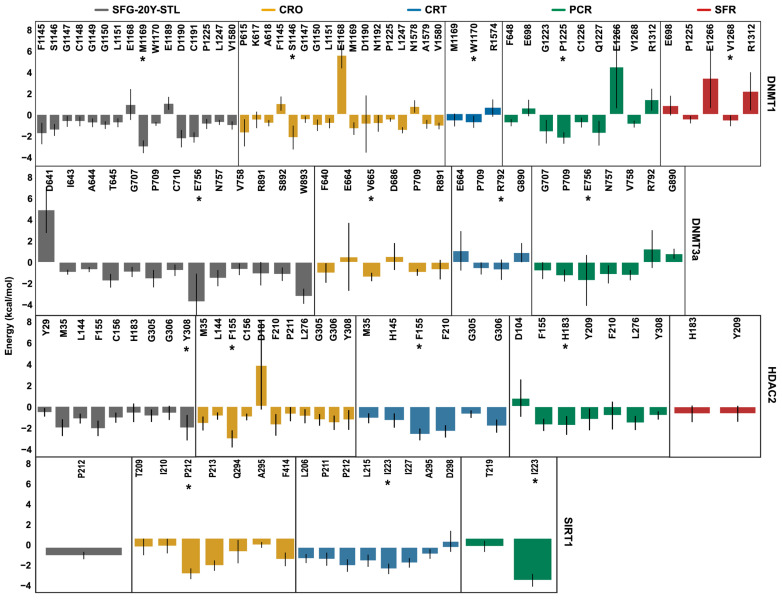
The per-residue MM/PBSA binding energy decomposition (kcal/mol) of the DNMT1, DNMT3a, HDAC2, and SIRT1 complexes. The marked residues (*) are those with high affinity (very low values of binding free energy).

To gain deeper insights into the binding mechanisms of the bioactive compounds under investigation, MM/PBSA per-residue energy decomposition analysis was performed on the ligand–target complexes and compared to the reference complex. Figure 7 provides a detailed molecular characterization of the interactions between the saffron-derived compounds and the epigenetic targets, highlighting the key residues involved in ligand binding, which are marked with stars. These residues are consistent with those identified by contact analysis (Table 3, Table 4, Table 5 and Table 6) and shown in Figure 3, Figure 4, Figure 5 and Figure 6 and Figure S7.

Taken together, these findings suggest that a combination of crocin, crocetin, and picrocrocin may exert a multi-target modulatory effect by simultaneously acting on DNMT1, HDAC2, and SIRT1 in a complementary manner.

### 2.4. In Silico Toxicity Evaluation of the Candidate Saffron Ligands and in Silico ADME Assessment

The toxicity analysis of the candidate saffron ligands, and for comparison of the control ligands, revealed significant variability in their predicted safety profiles (Table 8). Crocin, the main bioactive compound of saffron, emerged as the safest molecule among all tested ligands, showing no predicted adverse effects. It was classified as non-toxic (class VI) based on its high medial lethal dose (LD50) value (5600 mg/kg) and was found to be inactive against all organ-specific toxicity endpoints. Moreover, crocin showed no interaction with nuclear receptor or stress response pathways, further supporting its favorable toxicological profile. Among the other saffron-derived molecules, crocetin and safranal displayed moderate toxicity, with LD50 values of 4300 mg/kg and 5000 mg/kg, respectively, classifying them as potentially harmful (Class V). Picrocrocin, with an LD50 of only 55 mg/kg, was identified as the most toxic compound (class III). All the control ligands, sinefungin, 20Y, and resveratrol showed lower LD50 values ranging from 1000 to 1560 mg/kg and were considered as harmful (class IV). In terms of organ-specific toxicity, sinefungin showed multiple organ toxicities, being active for neurotoxicity, nephrotoxicity, and respiratory toxicity. Compound 20Y also exhibited a concerning profile with predicted hepatotoxic and neurotoxic effects and mutagenicity, while resveratrol was associated with nephrotoxicity and cardiotoxicity. Picrocrocin showed nephrotoxic and cardiotoxic properties, and safranal was predicted to be neurotoxic. The analysis of potential interactions with nuclear receptor pathways indicated limited activity across most control compounds. Notably, 20Y showed the activation of the aryl hydrocarbon receptor (AhR) and estrogen receptor (ER), while resveratrol activated the androgen receptor (AR), ER, and estrogen receptor ligand-binding domain (ER-LBD) pathways. All the saffron’s compounds showed no predicted interactions with the tested nuclear receptors, supporting their lower risk of endocrine disruption. Similarly, the evaluation of stress response pathways revealed activity only in a few compounds. Sinefungin and resveratrol were predicted to interfere with the mitochondrial membrane potential (MMP), and resveratrol was also active for ATAD5 (ATPase family AAA domain-containing protein 5), indicating potential involvement in the DNA damage response. Crocin remained inactive across all stress-related endpoints. It should be highlighted that the only synthetic compound analyzed (20Y ligand), although not classified as highly toxic based on LD50, showed the broadest impact across toxicity domains, including organs, nuclear receptors, and stress response pathways, and was also the only molecule predicted to have mutagenic potential.

The four saffron-derived compounds (crocin, crocetin, picrocrocin, and safranal) exhibited favorable ADME profiles (Table S2). Crocin and crocetin showed high gastrointestinal (GI) absorption, moderate lipophilicity, good solubility, and good blood–brain barrier (BBB) permeability, without being substrates of P-glycoprotein (P-gp). Safranal, the most lipophilic and synthetically accessible compound, also demonstrated excellent GI absorption and BBB penetration. Picrocrocin was characterized by its high polarity and solubility but lacked BBB permeability and was identified as a P-gp substrate. None of the saffron-derived molecules were predicted to inhibit major Cytochrome P450 (CYP450) enzymes, indicating a favorable metabolic profile. Compared to the controls, saffron compounds showed better compliance with drug-likeness rules and easier synthetic accessibility (except for picrocrocin).

Altogether, these findings highlight crocin as the safest compound among those analyzed, with no predicted toxicological concerns. In contrast, picrocrocin, in analogy to the sinefungin control for DNMTs, showed broad toxicity potential, underscoring the importance of thorough safety assessment before considering these molecules for therapeutic use.

## 3. Discussion

Despite the experimental and clinical evidence supporting the health benefits of saffron [52,53,54,55], the way its biocomponents can interact with human genome and specifically alter gene expression profile by epigenetic mechanisms has not been deeply elucidated.

Both DNA structures and nucleosome-related players have been preliminarily investigated as possible direct interactors of saffron molecules [15,56,57], and thus indirectly referred as epigenetic targets of the spice. The biological significance of such interaction has been mainly addressed in the context of cancer pathology and/or application of spice bioactives as promising new drugs [10,11,12].

By specifically focusing on key epigenetic enzymes, namely DNMTs and HDACs, we aimed at directly dissecting the epigenetic machinery and its involvement in the nutrigenomic/epigenomic properties of saffron. We first demonstrate that the impact of saffron phytochemicals varies according to the epigenetic target, supporting the selectively of their functionality.

Specifically, within the sinefungin-binding pocket, DNMT3a does not form stable interactions with any of the analyzed saffron bioactive compounds, whereas DNMT1 is bound and inhibited by beta-D-glucosyl trans-crocetin, one of the crocins of the saffron. Notably, this specific crocin engages key catalytic residues within the binding site in a manner that closely resembles the interaction pattern of sinefungin, which shares comparable chemical features and binding orientation. This structural and functional analogy reinforces the biological plausibility of crocin as a potential DNMT1 modulator.

To the best of our knowledge, these are the first data proving a direct interaction between a saffron-derived bioactive component and DNMTs, linking this food to the DNA methylation machinery. Several food-derived components, particularly those rich in polyphenols like green tea catechins and bioflavonoids, are known to inhibit DNMT1 activity [41,43]. Based on the increasing clinical interest in innovative antineoplastic “epi-drugs” that might silence DNMT function, the crocin inhibitory potential of DNMTs might be mainly exploited in terms of cancer therapy [58,59]. In this line, our in silico findings are supported by in vitro data reported by Khan et al. (2023) [60], where saffron treatment exerts antitumor effects in prostate cancer cell lines through the impairment of DNMT protein expression.

More intriguing, and still exhaustively unexplored, is the role of diet-induced DNMT inhibition in pathologies’ prevention and longevity. Age-dependent alterations in DNA methylation, including global hypomethylation and site-specific hypermethylation, have been linked to various age-related diseases, including cancer, diabetes, neurodegenerative disorders, and cardiovascular disease [61,62,63]. Therefore, targeting DNMTs with specific inhibitors—in order to delay or reverse these pathologies—has emerged as a potential anti-aging strategy. In this context, dietary genistein was shown to protect rats from chemically induced mammary tumors in a dose-dependent manner through the inhibition of DNMT activity; green tea epigallocatechin-3-gallate (EGCG) decreases DNMT protein levels and reduces 5-methyl cytosine content, thus exerting both cancer prevention and antidiabetic properties [64,65]. According to our computational analyses, crocin might play a similar role in preventing age-related pathologies and contributing to longevity via DNMT blocking. Consistent with this hypothesis, saffron has been already reported to drive a senescence-delaying effects in aging mice [66], to prevent skin aging induced by UV exposure [67], and to reduce age-related retinal degeneration in bright continuous light exposure rat models [68]; nevertheless, in vitro and in vivo studies are necessary to specifically address the saffron–DNMT hypothesis in aging.

In terms of chromatin remodeling, we tested the interaction of each saffron ligand with the HDAC2 enzyme, by screening the benzamide-binding pocket, where the inhibition of the enzymatic activity occurs. Our in silico analysis demonstrates that both crocin and crocetin strongly interact with HDAC2 to inhibit its activity. Certain dietary histone deacetylase inhibitors, such as curcumin, sulforaphane, EGCG, and resveratrol, have been proved to exert health protective effects; specifically, the inhibition of HDAC2 has been associated with the restoration of protein thiol redox homeostasis and protection against monocyte dysfunction induced by a high-calorie diet [69,70]. Experimental evidence suggests that the inhibition of HDAC2 may mitigate certain adverse effects associated with a high-calorie diet, including high-fat diet-induced hypertension and increased susceptibility to atherosclerosis. To date, *Crocus sativus* L-derived crocetin β-D-glucosyl ester has been demonstrated—via molecular docking analysis—to exhibit high affinity for HDAC2 and to inhibit breast cancer cell proliferation in vitro [71]. Our data confirm this result and provide new insights into the characterization of saffron biomolecules in the prevention of diet-related pathologic conditions via HDAC2 inhibition, paving the way for experimental and clinical validation based on our computational predictions. This finding is particularly noteworthy, as our data provide the first evidence that saffron affects HDAC2 activity, whereas previous research has focused exclusively on the modulation of HDAC1 and HDAC3 by saffron-derived compounds in human cancer cells [72,73].

Moreover, here, we demonstrate that crocin, crocetin, and picrocrocin interact and stimulate SIRT1; our data are in line with the docking evaluation performed by Krishnaswamy et al. (2020) [47] where crocetin and picrocrocin displayed the strongest binding energies with the enzyme, at levels comparable to the natural control activator, i.e., the resveratrol [49]. Our findings strengthen such interaction ability and extend—by the molecular dynamics approach—the functionality of such binding in terms of direct stimulation. Consistent with our data, Medoro et al. (2023) [48] reported that crocin has a high reactivity to SIRT1 and can form a stable complex with it, showing a good ability to fit into the binding pocket.

Similarly to many natural biomolecules, mainly resveratrol and curcumin that are reported to bind and activate SIRT1 as well as to drive SIRT1 expression levels [74,75], our computational prediction finds a first line of experimental validation in different in vitro and in vivo systems. Stimulation of SIRT1 by saffron phytochemicals has been experimentally tested in a breast cancer mice model undergoing 4 weeks of high-intensity interval training plus the administration of saffron aqueous extract, where the mRNA level of SIRT1 was found to be increased in double-treated animals if compared to control ones [76]. Although these data do not allow discrimination among individual saffron phytochemicals and support the ability of saffron to act on SIRT1 only when administered in combination with physical training, they provide an initial in vivo validation of our computational predictions. Furthermore, in patients with coronary artery disease (CAD) receiving crocin (30 mg/day), SIRT1 gene expression increased significantly in peripheral blood mononuclear cells if compared to placebo-treated ones [77]. In a mice model of repetitive mild traumatic brain injury (TBI), saffron extract was proved to attenuate the NOD-like receptors family pyrin domain-containing 3 (NLRP3) inflammasome signaling activation via the enhanced expression of SIRT1 in neuronal cells [78].

In addition, SIRT1 exerts broader regulatory functions than histone deacetylase, through its ability to tune downstream effectors, including FOXO (Forkhead box O), p53, and NF-κB (nuclear factor kappa B), thus playing a vital role in maintaining cellular health and longevity by influencing key processes like DNA repair, metabolism, and stress response. These SIRT1 targets amplify the biological effects of interactions of SIRT1 with diet-derived biomolecules.

In addition to their epigenetic modulatory potential and in the perspective of developing a saffron-based nutraceutical composition, we also evaluated the toxicological potential of the saffron-derived compounds. Crocin emerges as the safest candidate, showing no predicted adverse effects, including the absence of activation of nuclear receptor or stress response pathways, thereby supporting its favorable safety profile. Although picrocrocin displays a broader toxicity potential, all tested saffron-derived molecules notably do not activate nuclear receptor or stress-related pathways, suggesting a low risk of endocrine disruption or stress-mediated toxicity. In line with these findings, the favorable ADME properties of saffron-derived molecules further support their potential as bioavailable and safe bioactives. The lack of interaction with major CYP450 isoforms reduces the risk of metabolic drug–drug interactions, an important consideration for nutraceutical development. Moreover, the good GI absorption and BBB permeability of crocin, crocetin, and safranal may enhance their systemic efficacy, including potential central effects, while their non-inhibitory profile toward CYP450 enzymes aligns with a reduced metabolic liability. These pharmacokinetic and metabolic features, together with the low predicted toxicity, strengthen the case for crocin as a leading candidate in the formulation of saffron-based nutraceuticals.

While this study provides new insights into the potential epigenetic activity of saffron-derived compounds, it is important to acknowledge its limitations. All findings are derived exclusively from computational methods, including molecular docking, molecular dynamics simulations, and in silico toxicological approaches. Although these approaches offer valuable predictive information and mechanistic hypotheses, they cannot replace experimental validation. Therefore, further in vitro and in vivo studies will be essential to confirm the ability of these compounds to modulate epigenetic enzymes in a biological context and to assess their efficacy and bioavailability in physiological conditions.

In addition to the need for experimental validation, it is important to consider the potential for off-target interactions, especially with natural compounds known for their polypharmacological properties. Although a broad target screening was not performed in this study, our data provide preliminary evidence of selective binding behavior. Notably, beta-D-glucosyl trans-crocetin showed a stable and persistent interaction with DNMT1, but no significant binding was observed with DNMT3a, suggesting a certain degree of target specificity even within the same enzyme family. Furthermore, the ligand–residue contact analyses revealed that interactions occurred predominantly within the known binding regions of the target proteins, supporting a mechanistically meaningful binding mode. This is particularly evident for HDAC2 and SIRT1. These observations provide preliminary support for target selectivity and support the idea that the predicted interactions are not random or broadly promiscuous but potentially reflect biologically meaningful affinities compatible with subtle regulatory roles typical of dietary components. While these findings suggest specific binding, dedicated selectivity profiling and computational target prediction will be needed in future studies to fully assess the interaction specificity and mitigate the risk of off-target effects.

## 4. Materials and Methods

### 4.1. Protein Structures and Ligand Structures

This study focused on the human epigenetic enzymes DNMT1 (PDB ID: 3SWR), DNMT3a (PDB ID: 5YX2), HDAC2 (PDB ID: 4LY1, A chain), and SIRT1 (PDB ID: 5BTR, A chain). The 3D structures of DNMT1 and DNMT3A were obtained from our previous study [46], in which the structural gaps in the external loops of both crystal structures were accurately reconstructed. The crystal structure of sirtuin is a modified version of SIRT1 (SIRT1-143) that includes residues 143–665, but with amino acids 513–640 removed and the remaining parts joined together without an extra linker. This structure contains both the catalytic domain (CD) and the essential sirtuin-1 activity (ESA) region that are essential for deacetylase activity [49]. The missing atoms and residues (aa 141-143, 157-173, 659-665) in the PDB file of 5BTR structure were added by means of MODELLER code [79]. Among the 100 models generated, the one with the lowest DOPE score was selected and used as the most reliable structural model of SIRT1. A summary of DOPE scores is reported in the Supplementary Materials (Figure S9). The sequence alignment between the full-length FASTA sequence and the crystallographic structure of SIRT1 (PDB ID: 5BTR), along with the modeled structure highlighting the reconstructed regions, is provided in Supplementary Figure S10. In the ligand–receptor complexes involving the SIRT1 protein, the truncated form (RHK residues) of p53 substrate peptide was retained. The N-methyl (NME) capping group was added at the N-terminal, and the acetyl (ACE) capping group at the C-terminal of the RHK peptide.

The HDAC2 and DNMT1 enzymes are co-crystallized in a complex with their specific inhibitors. Specifically, HDAC2 is bound to a benzamide-class deacetylase inhibitor (4-(acetylamino)-N-[2-amino-5-(thiophen-2-yl)phenyl]benzamide) (20Y), while DNMT1 is complexed with sinefungin (SFG), its natural inhibitor. In the binding pocket of SIRT1, three resveratrol (STL) molecules are present. The structures of these ligands were extracted directly from the crystallographic data downloaded from the PDB and subsequently used as positive controls in re-docking studies and simulations. Among all the saffron molecules, the structures of the selected ligands, crocetin (CID 5281232) crocin (CID 10368299, beta-D-glucosyl crocetin), picrocrocin (CID 130796), and safranal (CID 61041), were searched in the PubChem database and downloaded as SDF files. The 2D chemical structures are shown in Figure S1. The identifier (ID) of each complex is reported in Table S3.

### 4.2. Molecular Docking

Molecular docking calculations were performed using AutoDock Vina [80,81]. The ligands downloaded from PubChem in the sdf format were converted to the pdb format by using the Open-Babel program [82]. Molecules were prepared by adding explicit hydrogen atoms and Kollman charges for the proteins and Gasteiger charges for the ligands by using AutoDockTools 1.5.6. As a positive control, the HDAC2 inhibitor (20Y), sinefungin (SFG), and resveratrol (STL, only one molecule of resveratrol was used) were re-docked, and the best pose obtained was compared to the co-crystallized one with HDAC2, DNMT1, and SIRT1, respectively. A semi-flexible docking approach was employed, in which the protein target was kept rigid while the ligand was treated as fully flexible. This approach ensures computational efficiency and adequate sampling of ligand conformations during the binding process. The grid boxes for HDAC2, SIRT1, and DNMTs targets were defined using the binding site of their co-crystallized ligands. For HDAC2 and SIRT1, the grid box dimensions were 45 × 45 × 45 Å, with centers at (x = 22.040, y = −17.680 and z = 1.680) and (x = −19.960, y = 61.320 and z = 11.680), respectively. DNMT1 and DNMT3A used grid box dimensions of 46 × 46 × 46 Å and 42 × 42 Å with center coordinates at (x = −4.282, y = −1.133, z = 32.921) and (x = 62.222, y = 32.553, z = −21.264), respectively. The Lamarckian genetic algorithm was used to scan the active site for low-energy-binding models and orientations. An ensemble of ten poses was generated for each docking run. The target–ligand complex with the lowest docking score (binding affinity) was selected from the ensemble and used as a starting configuration for the MD simulation. A summary of docking scores (kcal/mol) for all generated models is reported in Supplementary Materials (Table S4).

### 4.3. Molecular Dynamics Simulations

The target–ligand docking pose with the lowest binding energy was used as the starting configuration for the molecular dynamics (MD) simulation. All the simulations were performed by using GROMACS 2023.2 [83], with AMBER99sb-ildn force field for the receptors. The topology files for the ligands were generated using the ACPYPE (AnteChamber PYthon Parser interfacE) (https://www.bio2byte.be/acpype/submit/, accessed on 7 February 2024) [84]. The systems were placed in an octahedral box filled with TIP3P water model and neutralized using NaCl, which was added at the physiological concentration of 0.15 M. Periodic boundary conditions were applied to avoid edge effects. The energy of all the systems was minimized by using 5000 steps of steepest descent (SD), with a tolerance of 100 kJmol^−1^nm^−1^. The systems were gradually heated from 0 to 300 K and equilibrated using a protocol consisting of several steps, which are summarized in Table S5. After 300 ps of initial equilibration, MD simulations were carried out in the NPT ensemble for 200 ns, each step of 2 fs. The v-rescale algorithm, with a time constant of 0.1 ps, was used to keep the temperature at 300 K. The average pressure was kept at 1 bar with a time constant of 2 ps by using the isotropic Parrinello–Rahman barostat. The particle mesh Ewald (PME) method was used to compute the long-range electrostatics and van der Waals interactions with a cutoff of 1 nm. An equal cutoff was used for short-range electrostatics and van der Waals interactions. Rotation and translational motions of systems were removed, and all bonds were constrained by the LINCS algorithm. Initial velocities were assigned according to the Maxwell–Boltzmann distribution.

### 4.4. Trajectory Analysis

Trajectories were visualized using the VMD 1.9.4 software [85] and analyzed using tools available in the GROMACS package. Specifically, the root mean square deviations (RMSDs) and the root mean square fluctuations (RMSFs) were calculated by means of the *gmx rms* and *gmx rmsf* modules, respectively.

The *gromos* method was used in the clustering analysis by the *gmx cluster* tool. The *gromos* method was selected for its ability to identify dominant conformational families by grouping structures that are frequently sampled and geometrically similar. The algorithm constructs a matrix of pairwise RMSD values between all structures in the trajectory and identifies clusters based on a similarity criterion. Specifically, it identifies the structure with the largest number of neighboring structures (within a specified RMSD cutoff) as the center of the first cluster, assigns all the neighboring structures to it, removes them from the pool, and then iteratively repeats the process until all structures are assigned to a cluster. In this study, system-specific RMSD cutoffs are defined from the distribution of pairwise RMSD values (Figure S11). A threshold corresponding to the first local minimum in the distribution curve was selected, ensuring a balance between resolution and interpretability. This strategy helps avoid merging distinct conformers while minimizing excessive fragmentation of structurally similar states. The final RMSD cutoffs are as follows: 0.15 nm for DNMT1 systems, 0.18 nm for DNMT3A, 0.15 nm for HDAC2, and 0.30 nm for SIRT1 complexes.

The *gmx mindist* module was used to compute both the minimum distance and the number of close atomic contacts within 0.4 nm between any atom of the residues of the epigenetic target’s binding pocket and any atom of the ligands, in order to account for the widest range of potential non-covalent interactions, such as hydrogen bonds, van der Waals forces, hydrophobic contacts, salt bridges, and π-related interactions.

The *contactFreq.tcl* module in VMD was used to identify the target residues involved in interactions with the ligand using a distance cutoff of 0.4 nm. The module was also used to calculate the frequency of these contacts throughout the trajectory, expressed as the percentage of frames in which each contact was maintained, thereby providing a measure of interaction persistence.

The residence time of each ligand within the binding pocket was calculated using a geometric approach. In particular, the center-of-mass (COM) distance between each ligand and the binding site was computed from all the MD frames by using *gmx distance* module from GROMACS. The residence time (ns) was then defined as the number of frames in which this distance remained below a ligand-specific cutoff. To account for size variability among ligands, an adaptive cutoff was applied, corresponding to the maximum distance of any atom in the ligand from its own COM: 0.7 nm for SFG, CRO, and CRT ligands; 0.6 for PCR, 20Y, and STL ligands; and 0.35 for SFR ligand. This approach minimizes bias due to ligand size and ensures the consistent detection of binding events across compounds.

### 4.5. MM/PBSA Calculations

The MM/PBSA (Molecular Mechanics/Poisson–Boltzmann Surface Area) method was employed to calculate binding free energies between target proteins and ligands. This approach combines molecular mechanics energy terms with implicit solvation models to estimate the free energy of binding [86]. The average free energies of solvation (ΔG_binding_) between target proteins and the set of ligands can be obtained by Equation (1):

ΔG_binding_ = G_complex_ − G_protein_ − G_ligand_ (1)

where G_complex_ is the total free energy of the protein–ligand complex, and G_protein_ and G_ligand_ are the total free energies of the separated protein and ligand in solvent, respectively. The single-trajectory MM/PBSA protocol, where both the target and ligand trajectories are derived from the complex trajectory, was employed in this study to save computational costs. This approach is justified by the high structural similarity between the target conformations in the unbound (apo) and bound states, as indicated by average RMSD values below 0.25 nm [46]. The binding free energy was, therefore, calculated by Equation (2):

ΔG_binding_ = E_MM_ + G_solv_ − T⋅S (2)

where E_MM_ corresponds to the molecular mechanics-derived energy changes in the gas phase, and includes internal energy (E_bonded_) and van der Waals (E_vdW_) and electrostatic contributions (E_ele_) as illustrated in Equation (3):

E_MM_ = E_bonded_ + E_non-bonded =_ E_bond_ + E_angle_ + E_dihedral_ + (E_ele_ + E_vdW_) (3)

In Equation (2), G_solv_ is the solvation energy, and T⋅S is the entropic contribution of the solute. The entropic in Equation (4) can be estimated using normal mode analysis; however, it was omitted in our study. This decision was based on the aim to compare states with similar entropy, such as the different ligands binding to the same protein. Furthermore, normal mode analysis is computationally demanding and susceptible to considerable margins of error, which can lead to significant uncertainty in the results. The solvation energy G_solv_ is composed by two terms G_polar_ and G_nonpolar_ that refer to electrostatic and non-electrostatic contributions, as illustrated in Equation (4):

G_solv_ = G_polar_ + G_non-polar_ (4)

The first term is obtained by solving Poisson–Boltzmann equations, whereas the non-polar solvation energy is separated into cavity and dispersion terms. In this approach, the molecule’s total solvent accessible surface area (SASA) is used to correlate the cavity term, while a surface-based integration method is employed to compute the dispersion term Equation (5):

G_non-polar_ = G_disp_ + G_cavity_ = G_disp_ + (CAVITY_TENSION_ × SASA + CAVITY_OFFSET_) (5)

The average of the binding free energy and of all its components of all the ligand–target complexes was calculated by using the gmx-MMPBSA v1.6.4 tool [87] for the full trajectory. The residues of the binding site, which have a main role in the interaction with ligands, were also identified using the MM/PBSA free energy decomposition analysis.

### 4.6. Toxicity Prediction of the Ligands

The toxicity prediction of the ligands (control and saffron compounds) was carried out using ProTox 3.0 (https://tox.charite.de/protox3/index.php?site=compound_input/, accessed on 28 April 2025) [88], a tool that combines molecular similarity and machine learning models to evaluate various toxicity endpoints. The analysis includes oral toxicity (acute toxicity in rodents), organ toxicity (such as hepatotoxicity), general toxicological effects (including mutagenicity, carcinogenicity, cytotoxicity, and immunotoxicity), and molecular initiating events and adverse outcome pathways (AOPs), providing insights into the possible molecular mechanisms behind toxic responses. In particular, the compounds were screened to assess their potential to interfere with biological pathways, focusing on two major outcome categories: nuclear receptor signaling and stress response pathways. Within the nuclear receptor signaling group, seven target-based models were considered, i.e., aryl hydrocarbon receptor (AhR), androgen receptor (AR), androgen receptor ligand-binding domain (AR-LBD), aromatase, estrogen receptor alpha (ER), estrogen receptor ligand-binding domain (ER-LBD), and peroxisome proliferator-activated receptor gamma (PPAR-γ). For stress response pathways, five models were included, i.e., nuclear factor (erythroid-derived 2)-like 2 (NRF2)/antioxidant response element (ARE), heat shock factor response element (HSE), mitochondrial membrane potential (MMP), tumor suppressor protein p53, and ATPase family AAA domain-containing protein 5 (ATAD5). Compounds are classified into six toxicity classes based on the Globally Harmonized System (GHS), using the median lethal dose (LD50), which represents the dose required to cause death in 50% of test organisms. Class I includes compounds with LD50 ≤ 5 mg/kg and is considered extremely toxic. Class II includes those compounds with LD50 between 5 and 50 mg/kg and is also considered highly toxic. Class III (LD50 between 50 and 300 mg/kg) is classified as toxic, while Class IV (LD50 between 300 and 2000 mg/kg) is considered harmful. Class V (LD50 between 2000 and 5000 mg/kg) includes compounds that may be harmful, and Class VI (LD50 > 5000 mg/kg) includes non-toxic substances.

### 4.7. ADME Profile of the Ligands

The ADME profile of the analyzed compounds was evaluated in silico using the SwissADME online platform (http://www.swissadme.ch/, accessed on 20 July 2025), developed by the Swiss Institute of Bioinformatics [89]. Based on the canonical SMILES representations of the molecules, the tool computed key pharmacokinetic and physicochemical properties, including drug-likeness and lead-likeness. Drug-likeness was assessed through multiple rule-based filters—namely, Lipinski’s rule of five, the Ghose filter, Veber rules, Egan rules, and Muegge rules—to predict oral bioavailability and other drug-like characteristics.

## 5. Conclusions

The integration of molecular docking and molecular dynamics simulations played a key role in accurately assessing the stability and relevance of interactions between saffron-derived compounds (crocin, crocetin, picrocrocin, and safranal) and epigenetic targets (DNMT1, DNMT3a, HDAC2, and SIRT1). This combined approach enabled us to distinguish transient contacts from potentially functional bindings. Our results reveal a complementary interaction profile suggestive of potential synergistic effects of the compounds. Crocin emerges as a promising DNMT1 inhibitor and, along with crocetin, also shows the ability to inhibit HDAC2 and activate SIRT1. None of the compounds displays stable binding to DNMT3a. Although safranal shows weak affinity for all targets, its known antioxidant and anti-inflammatory properties may still contribute to a favorable epigenetic environment. This pattern of mild, multi-target modulation aligns with the concept of functional foods, where long-term benefits derive from regular dietary intake rather than pharmacological dosing. Moreover, toxicity predictions indicate that all the saffron compounds, here considered, are non-toxic or with low toxicity, supporting their safety for regular consumption.

Overall, the data support the potential of saffron as a safe, naturally balanced source of epigenetically active molecules, capable of contributing to epigenetic homeostasis and health maintenance. With these results being predictive and based solely on in silico modeling, functional experimental validation remains essential to confirm the observed interactions and to evaluate their specificity and efficacy. Nonetheless, our findings pay the way for considering saffron not just as a spice but also as a functional food with epigenetic potential. The identification of specific bioactive compounds provides a starting point for developing translational strategies, including saffron-based nutraceuticals or therapeutic formulations targeting age-related and chronic diseases through epigenetic modulation. Furthermore, the most promising ligand–target complexes could benefit from further investigation using more rigorous methods, such as free energy perturbation (FEP) or metadynamics, to enhance prediction accuracy and guide experimental validation.

## Figures and Tables

**Table 1:** Molecular docking results. Predicted binding affinity—docking score (kcal/mol)—obtained by the empirical scoring function of AutoDock Vina of the best docked complexes.

Ligand	DNMT1	DNMT3a	HDAC2	SIRT1
**SFG/20Y/STL ^(a)^**	−9.3	−8.1	−8.4	−8.0
**CRO ^(b)^**	−8.9	−9.2	−7.3	−8.5
**CRT**	−7.8	−8.3	−7.5	−8.1
**PCR**	−8.2	−7.2	−6.1	−7.3
**SFR**	−6.0	−5.2	−5.6	−5.2

^(a)^ SFG, sinefungin: an inhibitor of DNMT1 and DNMT3a; 20Y, 4-(acetylamino)-N-[2-amino-5-(thiophen-2-yl) phenyl] benzamide: an inhibitor of HDAC2; STL, resveratrol molecule: an activator of SIRT1. ^(b)^ CRO, crocin; CRT, crocetin-1; PCR, picrocrocin; SFR, safranal.

**Table 2:** The average number of close atomic contacts (<0.4 nm) between the binding pocket’s residues of the receptor and the atoms of the ligand. The time evolution of the number of these contacts is shown in Figure S5.

*Ligand*	DNMT1	DNMT3a	HDAC2	SIRT1
**SFG/20Y/STL ^(a)^**	463.60 ± 25.82	367.27 ± 31.44	149.04 ± 20.29	74.56 ± 22.52
**CRO ^(b)^**	371.31 ± 30.59	174.17 ± 50.88	241.10 ± 24.48	202.24 ± 30.84
**CRT**	62.62 ± 39.87	157.67 ± 55.52	192.74 ± 19.97	144.34 ± 27.16
**PCR**	167.31 ± 18.01	119.02 ± 34.71	137.67 ± 32.20	121.69 ± 18.66
**SFR**	43.44 ± 17.29	9.11 ± 25.27	19.00 ± 19.20	6.08 ± 15.65

^(a)^ SFG, sinefungin: an inhibitor of DNMT1 and DNMT3a; 20Y, 4-(acetylamino)-N-[2-amino-5-(thiophen-2-yl) phenyl] benzamide: an inhibitor of HDAC2; STL, resveratrol molecule, an activator of SIRT1. ^(b)^ CRO, crocin; CRT, crocetin-1; PCR, picrocrocin; SFR, safranal. Average and standard deviation values are reported.

**Table 7:** Binding free energies (ΔG, kcal/mol), predicted by MM/PBSA, of DNMT1-, DNMT3a-, HDAC2-, and SIRT1-binding site in a complex with ligands. Bold text indicates very low values of binding free energy (high affinity).

Ligand	DNMT1	DNMT3a	HDAC2	SIRT1
**SFG/20Y/STL^(a)^**	**−47.17 ± 6.64**	**−42.82 ± 9.22**	**−42.48 ± 5.96**	**−25.80 ± 2.66**
**CRO ^(b)^**	**−42.39 ± 8.24**	−21.96 ± 7.43	**−45.00 ± 8.14**	**−50.17 ± 4.50**
**CRT**	−21.31 ± 6.38	−21.47 ± 4.53	**−47.23 ± 4.12**	**−34.49 ± 5.62**
**PCR**	−25.69 ± 7.66	−20.03 ± 7.56	−25.67 ± 5.70	−23.31 ± 2.15
**SFR**	−9.8 ± 4.50	−3.61 ± 4.17	−14.42 ± 6.01	−16.51 ± 3.16

^(a)^ SFG, sinefungin: an inhibitor of DNMT1 and DNMT3a; 20Y, 4-(acetylamino)-N-[2-amino-5-(thiophen-2-yl) phenyl] benzamide: an inhibitor of HDAC2; STL, resveratrol molecule, an activator of SIRT1. ^(b)^ CRO, crocin; CRT, crocetin-1; PCR, picrocrocin; SFR, safranal. Standard deviation values are reported.

**Table 8:** Toxicity analysis. Bold text indicates a potential toxicity concern.

	SFG	20Y	STL	CRO	CRT	PCR	SFR
**Predicted Toxicity Class**							
LD50 (mg/kg)	1000	1500	1560	5600	4300	55	5000
Class	IV	IV	IV	VI	V	III	V
**Organ Toxicity**							
Hepatotoxicity	Inactive	**Active**	Inactive	Inactive	Inactive	Inactive	Inactive
Neurotoxicity	**Active**	**Active**	Inactive	Inactive	Inactive	Inactive	**Active**
Nephrotoxicity	**Active**	Inactive	**Active**	Inactive	**Active**	**Active**	Inactive
Respiratory toxicity	**Active**	Inactive	Inactive	Inactive	Inactive	Inactive	Inactive
Cardiotoxicity	Inactive	Inactive	**Active**	Inactive	Inactive	**Active**	Inactive
**Endpoint Toxicity**							
Carcinogenicity	Inactive	Inactive	Inactive	Inactive	Inactive	Inactive	Inactive
Immunotoxicity	Inactive	Inactive	Inactive	Inactive	Inactive	Inactive	Inactive
Mutagenicity	Inactive	**Active**	Inactive	Inactive	Inactive	Inactive	Inactive
Cytotoxicity	Inactive	Inactive	Inactive	Inactive	Inactive	Inactive	Inactive
**Nuclear Receptor Pathway**							
AhR	Inactive	**Active**	Inactive	Inactive	Inactive	Inactive	Inactive
AR	Inactive	Inactive	Active	Inactive	Inactive	Inactive	Inactive
AR-LBD	Inactive	Inactive	Inactive	Inactive	Inactive	Inactive	Inactive
Aromatase	Inactive	Inactive	Inactive	Inactive	Inactive	Inactive	Inactive
ER	Inactive	**Active**	**Active**	Inactive	Inactive	Inactive	Inactive
ER-LBD	Inactive	Inactive	**Active**	Inactive	Inactive	Inactive	Inactive
PPAR-Gamma	Inactive	Inactive	Inactive	Inactive	Inactive	Inactive	Inactive
**Stress Response Pathway**							
nrf2/ARE	Inactive	Inactive	Inactive	Inactive	Inactive	Inactive	Inactive
HSE	Inactive	Inactive	Inactive	Inactive	Inactive	Inactive	Inactive
MMP	Inactive	**Active**	**Active**	Inactive	Inactive	Inactive	Inactive
p53	Inactive	Inactive	Inactive	Inactive	Inactive	Inactive	Inactive
ATAD5	Inactive	Inactive	**Active**	Inactive	Inactive	Inactive	Inactive

## Data Availability

The dataset is available on request from the authors.

## Supplementary Materials

The following supporting information can be downloaded at: https://www.mdpi.com/article/10.3390/ijms26157575/s1.

## Abbreviations

The following abbreviations are used in this manuscript:

20Y	4-(acetylamino)-N-[2-amino-5-(thiophen-2-yl) phenyl] benzamide
ACE	acetyl group
ACPYPE	AnteChamber PYthon Parser interfacE
AhR	aryl hydrocarbon receptor
AOP	adverse outcome pathways
AR	androgen receptor
AR-LBD	androgen receptor ligand-binding domain
ATAD5	ATPase family AAA domain-containing protein 5
BBB	Blood–brain barrier
CD	catalytic domain
CR	caloric restriction
CRO	beta-D-glucosyl trans-crocetin, crocin
CRT	crocetin
CYP450	Cytochrome P450
DNMT	DNA methyl transferase
EGCG	green tea epigallocatechin-3-gallate
ER	estrogen receptor alpha
ER-LBD	estrogen receptor ligand-binding domain
ESA	essential sirtuin-1 activity
FOXO	Forkhead box O
GHS	Globally Harmonized System
GI	gastrointestinal
HA	histone acetylation
HAT	histone acetyl transferase
HDAC	histone deacetylase
HSE	heat shock factor response element
LD50	median lethal dose
MD	molecular dynamics
MMP	mitochondrial membrane potential
MM/PBSA	Molecular Mechanics/Poisson–Boltzmann Surface Area
NF-Kb	nuclear factor kappa-light-chain-enhancer of activated B cells
NLRP3	NOD-like receptors family pyrin domain-containing 3
NME	N-methyl group
nrf2/ARE	nuclear factor (erythroid-derived 2)-like 2/antioxidant response element
NTD	N-terminal domain
p53	tumor suppressor protein
P-gp	P-glycoprotein
PCR	picrocrocin
PME	particle mesh Ewald
PPAR-γ	peroxisome proliferator-activated receptor gamma
PTM	post-translational modification
RMSD	root mean square deviation
RMSF	root mean square fluctuation
SD	steepest descent
SFR	safranal
STL	resveratrol
TBI	traumatic brain injury
