# Supplementary material for: Epigenomics Nutritional Insights of Crocus sativus L.: Computational Analysis of Bioactive Molecules Targeting DNA Methyltransferases and Histone Deacetylases
Source: Int J Mol Sci. 2025 Aug 5;26(15):7575. doi: 10.3390/ijms26157575 (PMC12347544; doi:10.3390/ijms26157575)
Supplement: Supplementary file 1 [file ijms-26-07575-s001.zip › ijms-3737607-supplementary.pdf]

International Journal of *Molecular Sciences*

MDPI

SUPPLEMENTARY MATERIAL

Epigenomic Nutritional Insights of *Crocus sativus* L.: Computational Analysis of Bioactive Molecules and Their Interaction with Epigenetic Targets

Alessia Piergentili [1,2,†], Paolo Roberto Saraceni [3,†], Olivia Costantina Demurtas [3], Barbara Benassi [3,*] and Caterina Arcangeli [3,*]

[1] Institute for Neuroscience and Medicine (INM-9), Forschungszentrum Jülich, Wilhelm-Johnen-Straße, 52428 Jülich, Germany; a.piergentili@fz-juelich.de
[2] Department of Neurology, University Hospital Aachen, RWTH Aachen, Pauwelsstraße 30, 52074 Aachen, Germany
[3] Department for Sustainability, Italian National Agency for New Technologies, Energy and Sustainable Economic Development (ENEA) Casaccia Research Center, Via Anguillarese 301, 00123 Rome, Italy; paolo.saraceni@enea.it (P.R.S.); olivia.demurtas@enea.it (O.C.D.)
* Correspondence: barbara.benassi@enea.it (B.B.); caterina.arcangeli@enea.it (C.A.)
† The authors contributed equally to this work.

Figure S1 – 2D Chemical Structure
Figure S2 – RMSD Analysis
Figure S3 – RMSF Analysis
Figure S4 – Distance Analysis
Figure S5 – Contact Analysis
Figure S6 – Visual Inspection of Control Complexes
Figure S7 – Close-up view of the Ligand-Target Binding Interface
Figure S8 – Binding Free Energy Analysis
Figure S9 - DOPE Scores of Homology Models
Figure S10 - Sequence Alignment and Homology Model of SIRT1
Figure S11 – Distribution of pairwise RMSD values.
Table S1 – Cluster Analysis
Table S2 – In silico ADME analysis
Table S3 – Ligand-Target Complexes
Table S4 - Docking pose stability analysis
Table S5 – Equilibration Procedure.

https://doi.org/10.3390/xxxxx

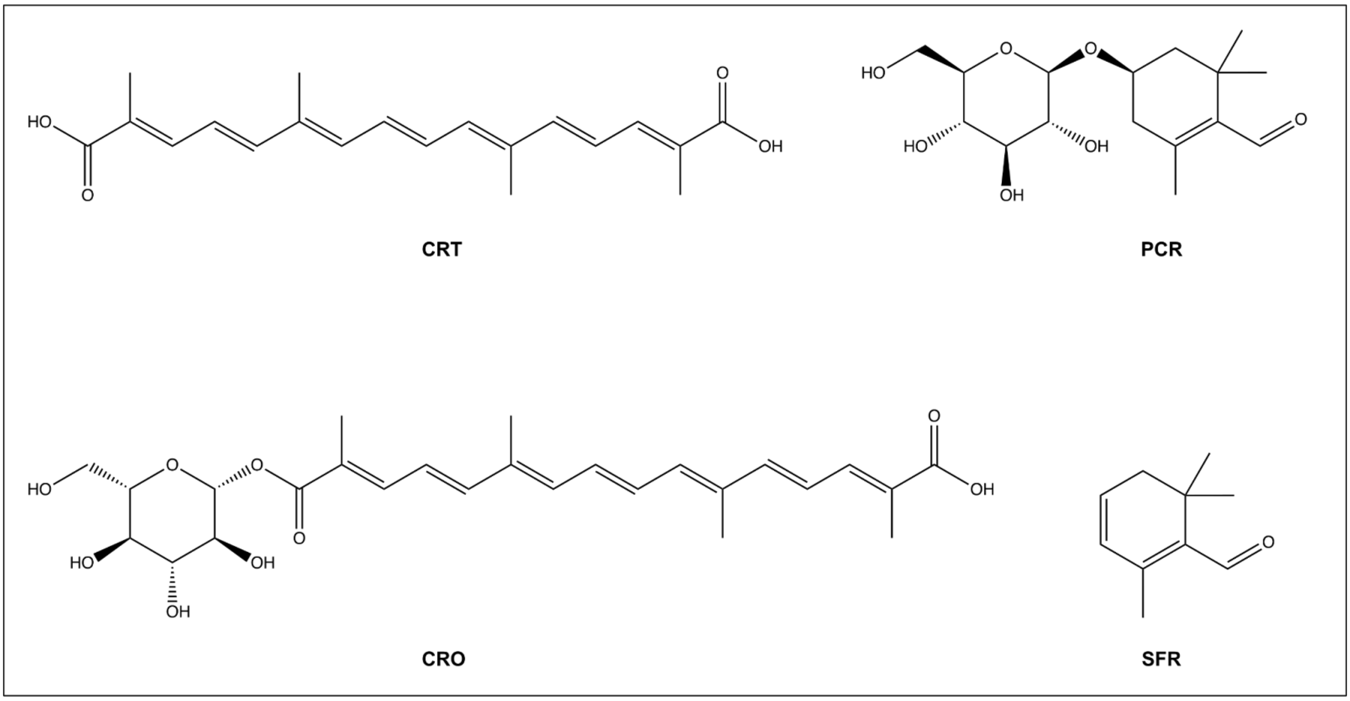

Figure S1 – 2D Chemical Structures. The 2D chemical structure of the screened saffron's biomolecules: Crocetin (CRT), beta-D-glucosyl trans crocetin (CRO), Picrocrocin (PCR), Safranal (SFR).

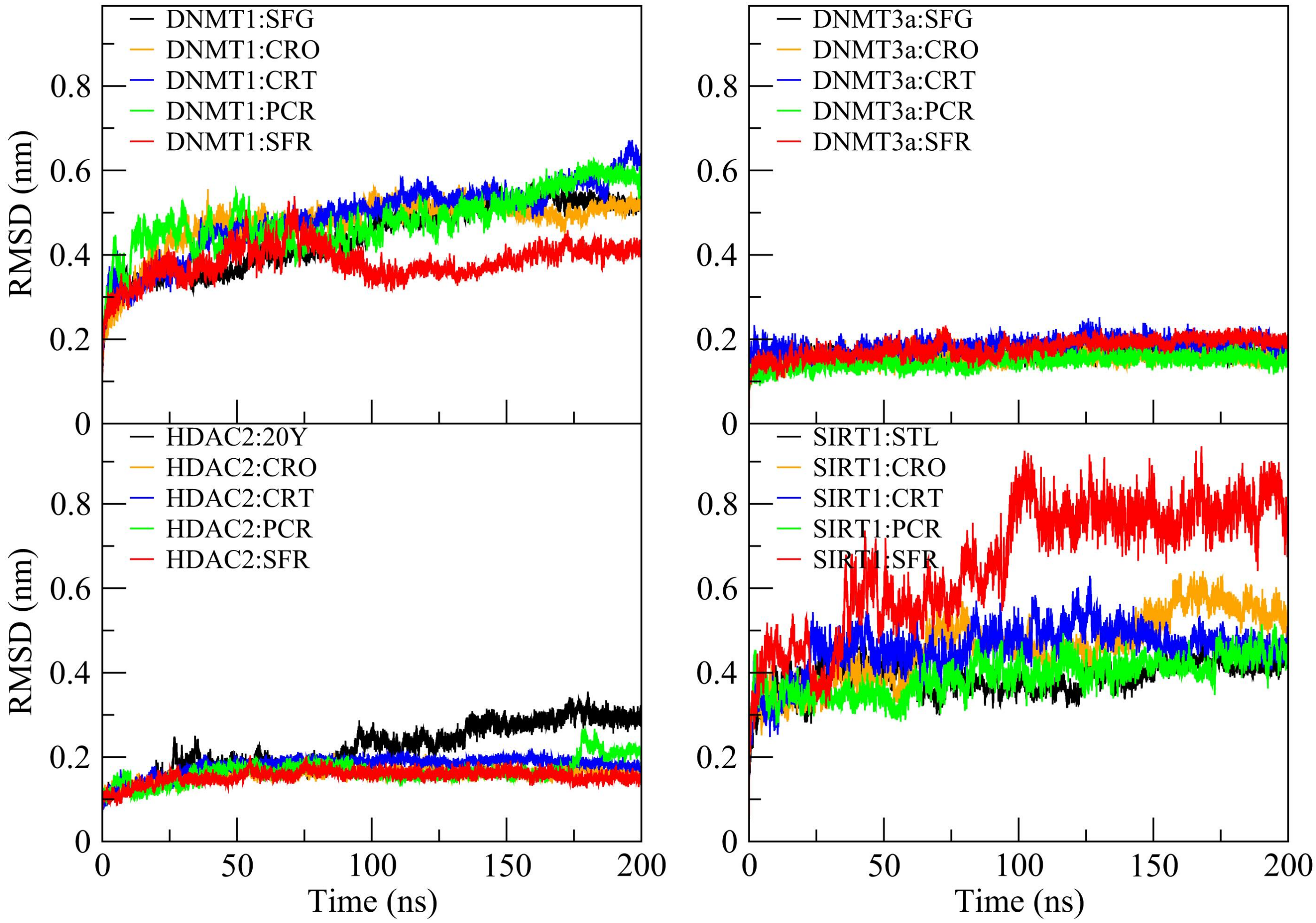

Figure S2 – RMSD Analysis. RMSD's values of the Cα atoms of the epigenetic targets in complex with ligands, as a function of simulation time.

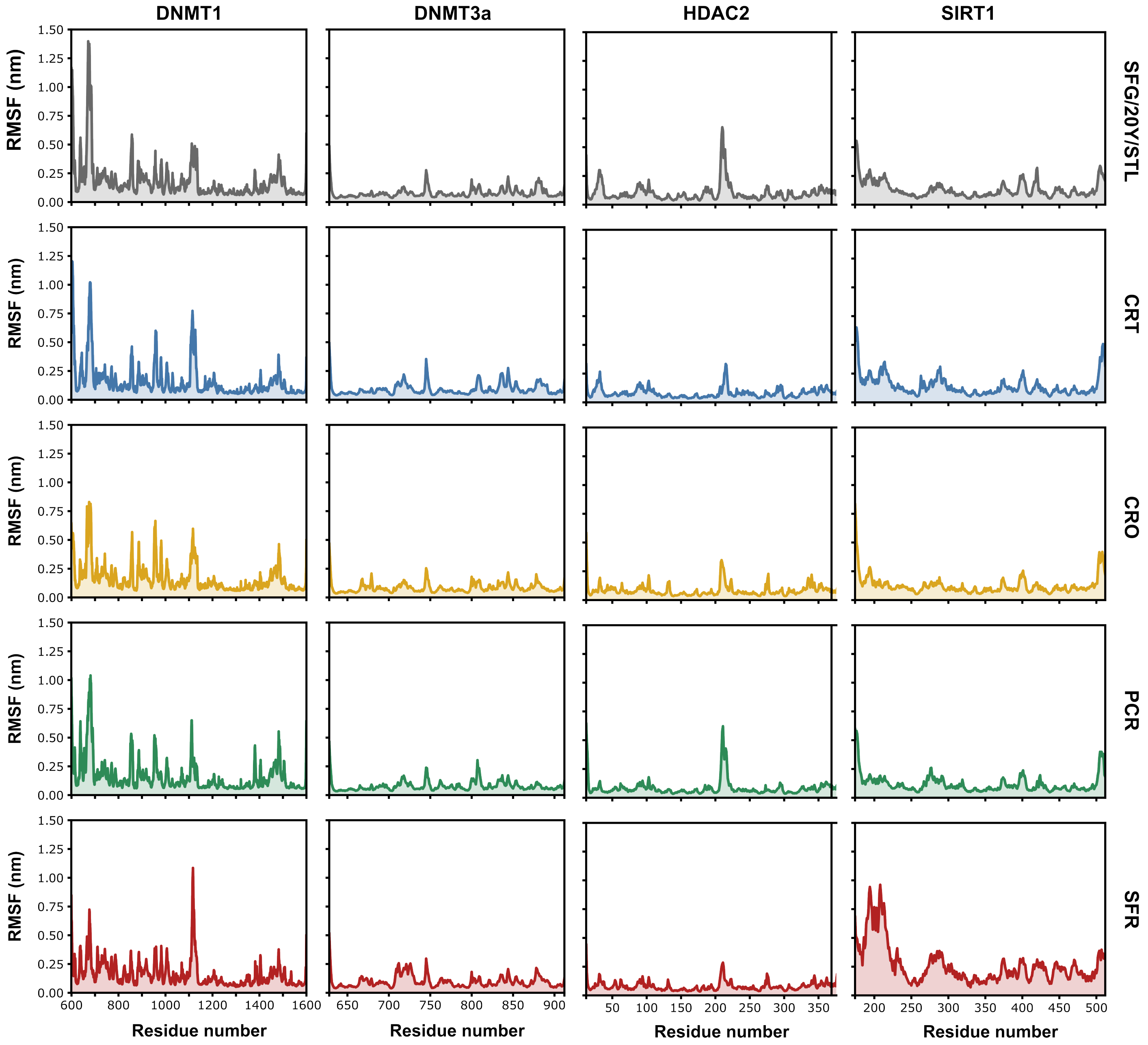

Figure S3 – RMSF Analysis. RMSF's values of the Cα atoms of the epigenetic targets in complex with ligands, as a function of the residues.

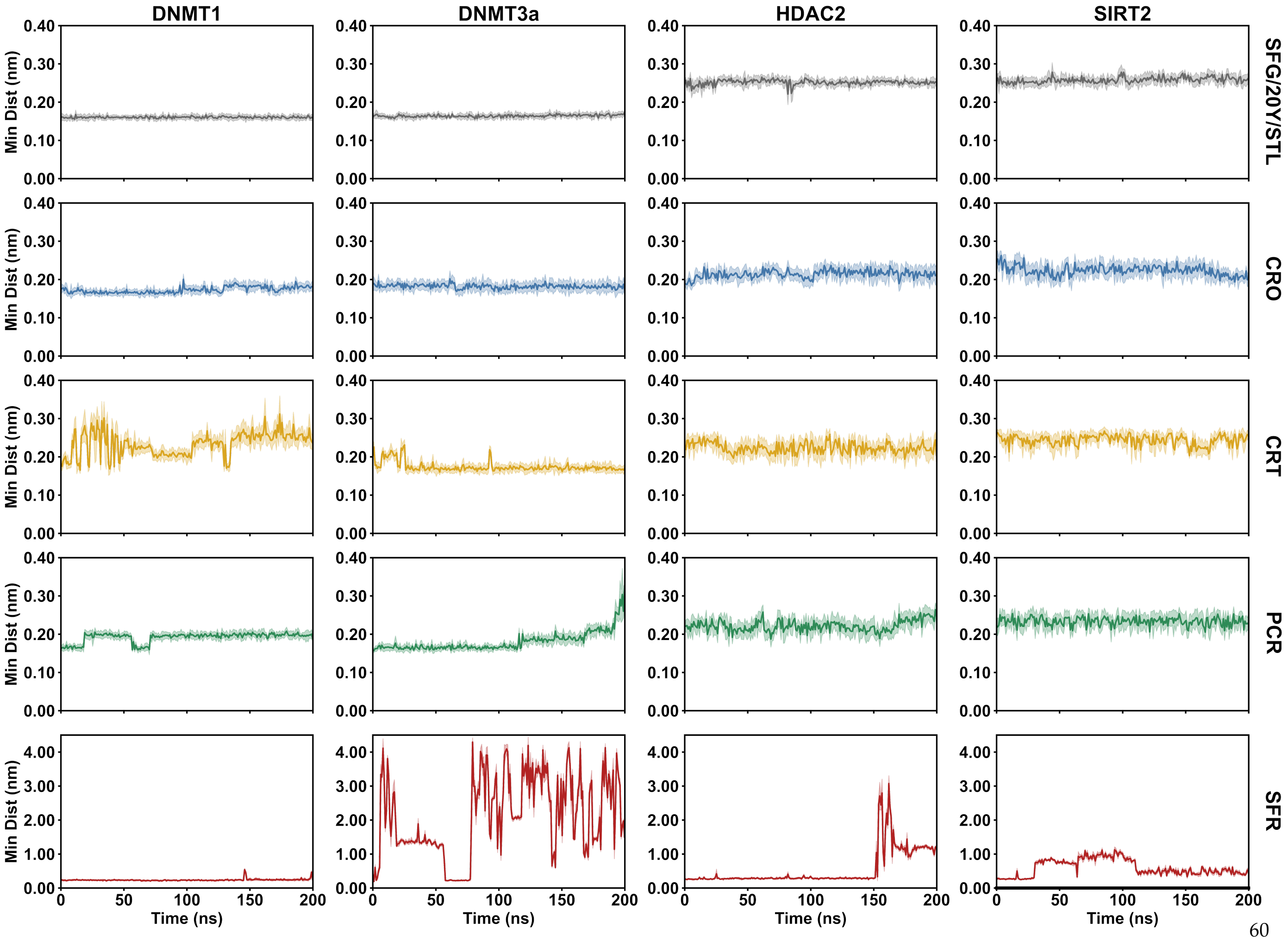

Figure S4 – Minimum Distance Analysis. Time evolution of minimum distance (nm) between ligands and binding pocket residues in DNMT1, DNMT3a, HDAC and SIRT1 system.

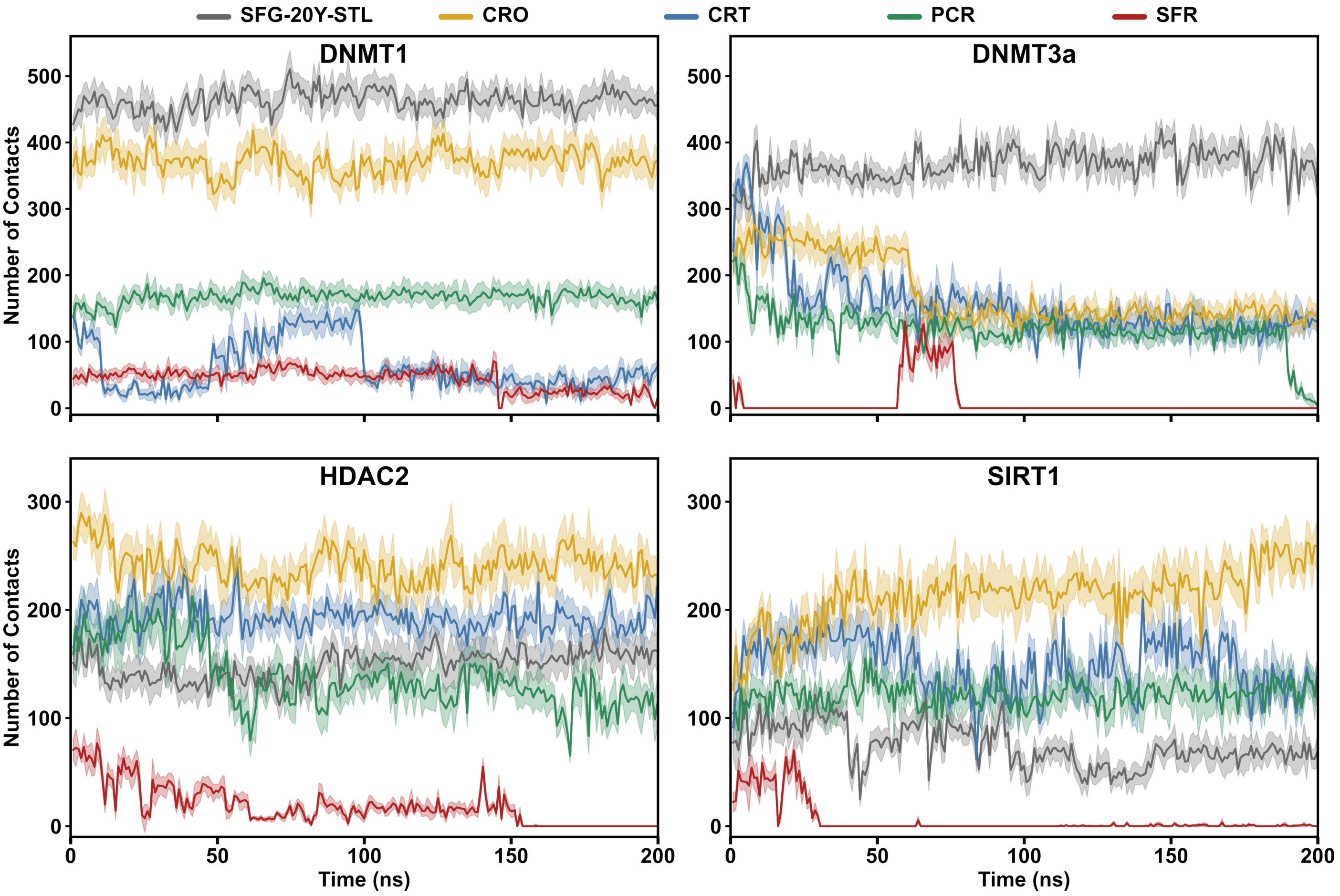

Figure S5 – Contact Analysis. Time evolution of atomic contacts (<0.4 nm) between ligands and binding pocket residues in DNMT1, DNMT3a, HDAC and SIRT1 systems.

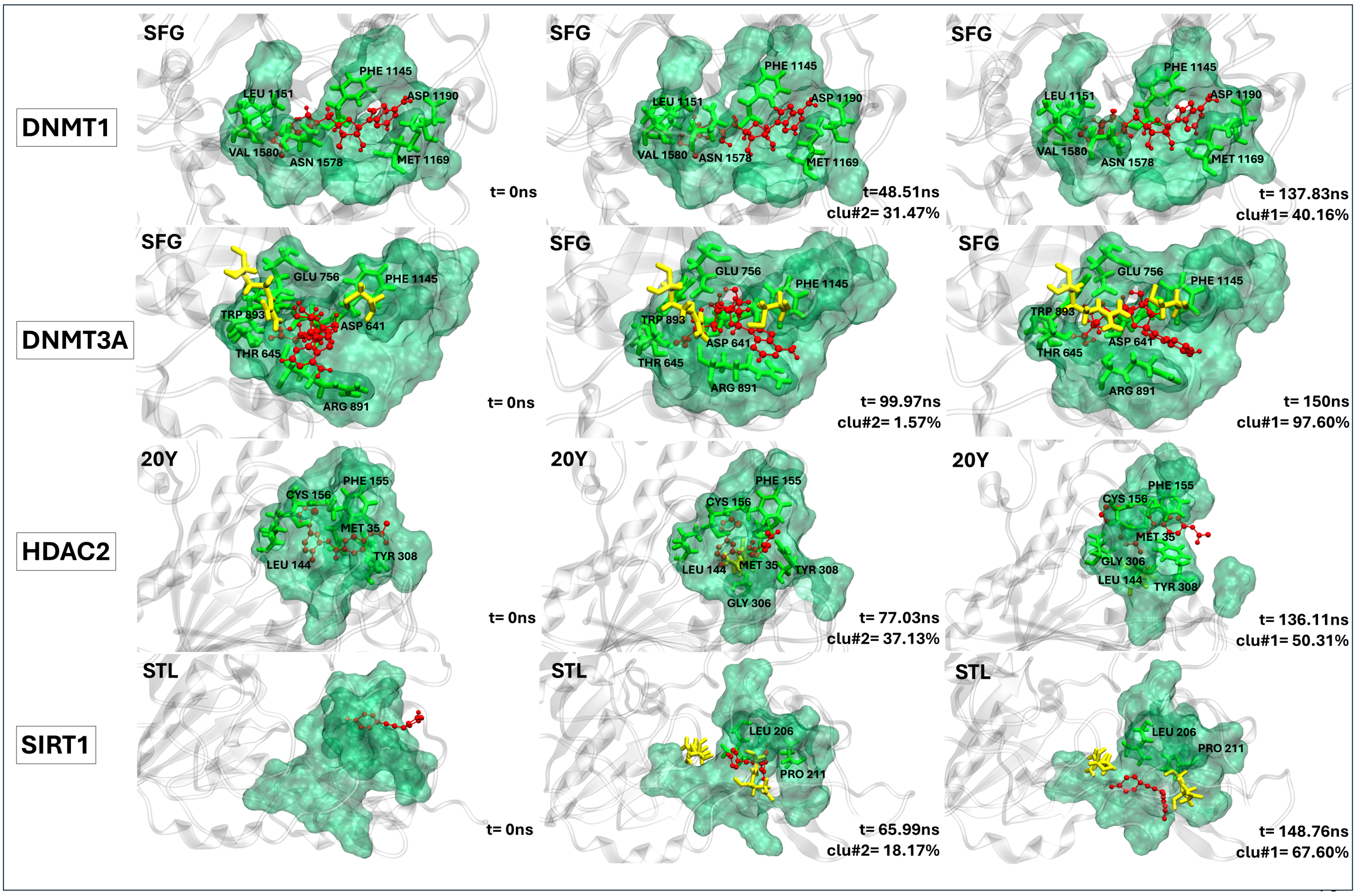

Figure S6. Representative structures of MD trajectory extracted from the first frame (t=0 ns and the two most populated clusters of the control complexes: DNMT1:SFG, DNMT3a:SFG, HDAC2:20Y and SIRT1:STL. Binding pocket residues are colored in green, extra binding pocket residues in yellow, and ligands in red.

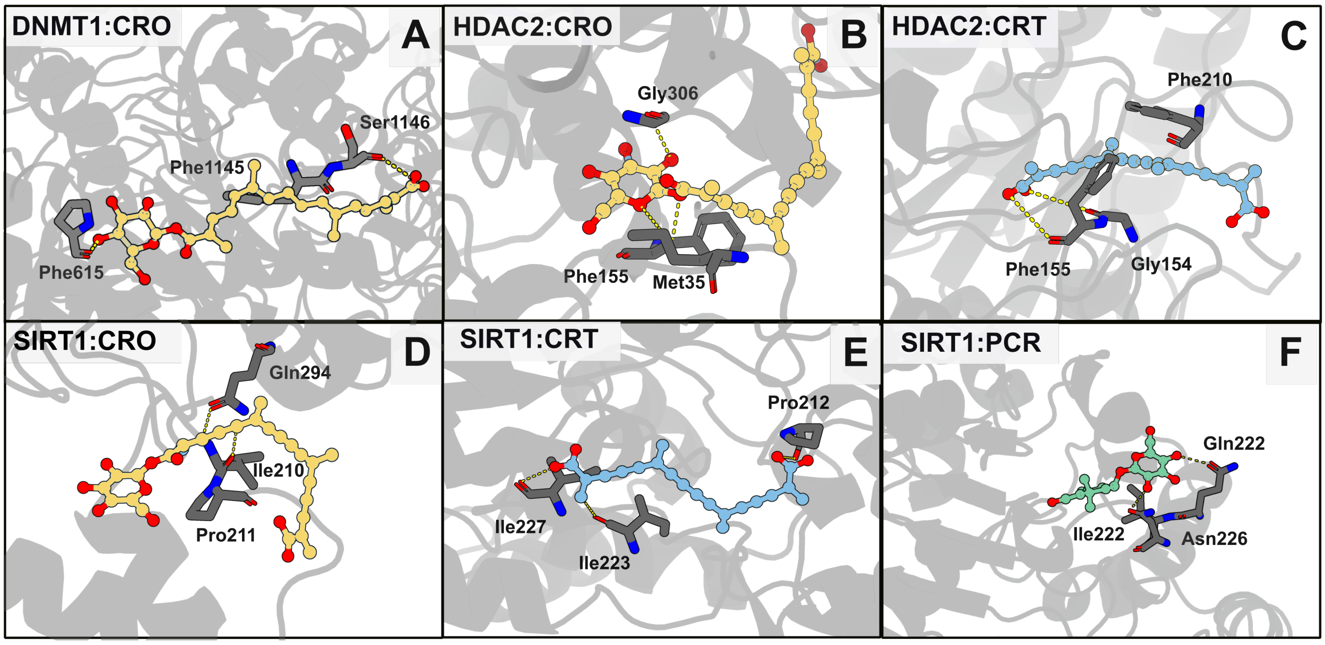

Figure S7. Close-up views of the ligand–target binding interfaces, illustrating key interactions and atomic distances for the following complexes: (A) DNMT1:CRO, (B) HDAC2:CRO, (C) HDAC2:CRT, (D) SIRT1:CRO, (E) SIRT1:CRT, and (F) SIRT1:PCR. Residues in close contact with the ligands are displayed. Dashed lines represent interactions within 4 nm between binding pocket residues and the ligands.

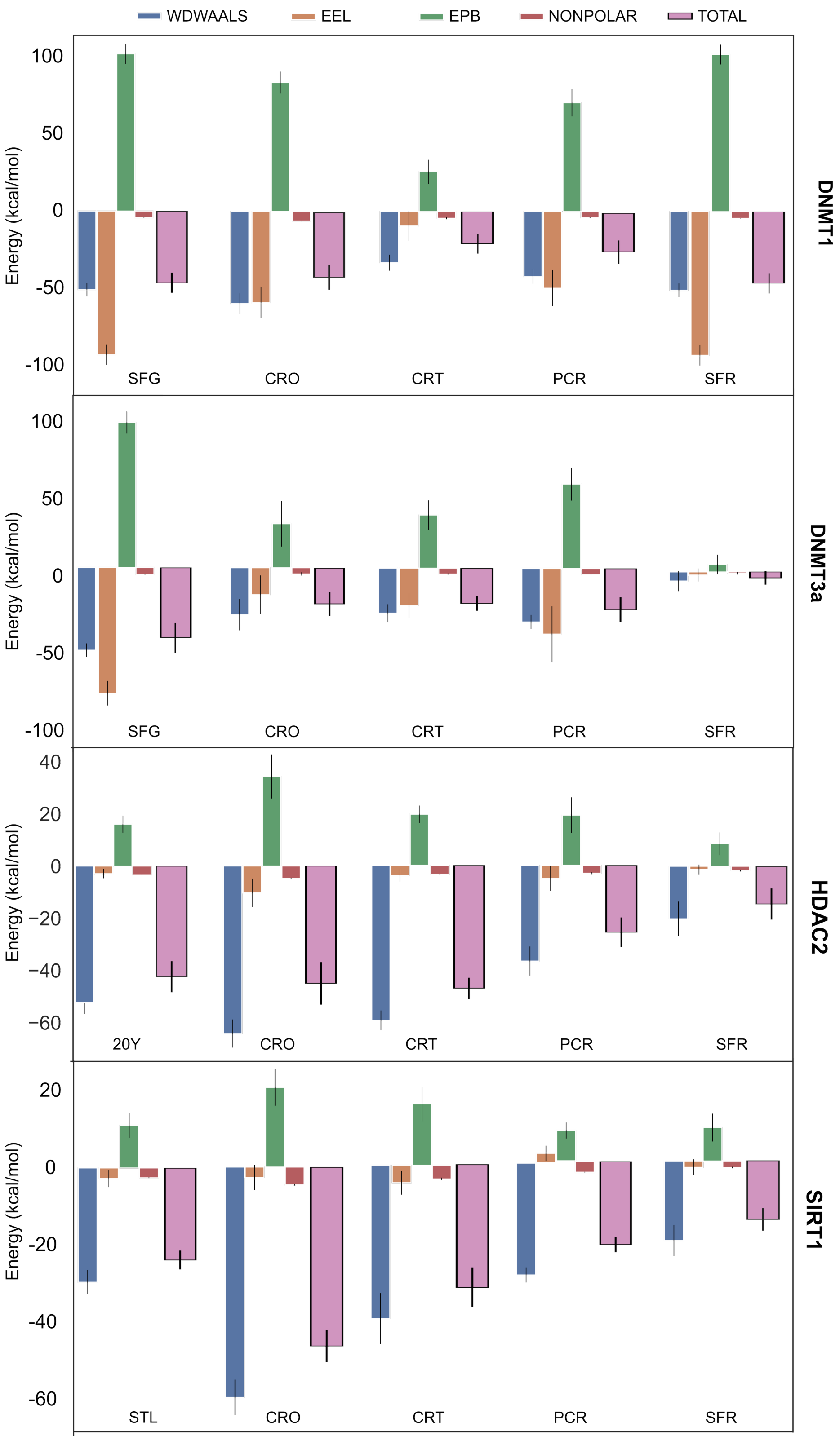

Figure S8 – Binding Free Energy Analysis. Binding energy calculation of the ligands with the epigenetic targets. VDWAALS: van der Waals contribution from MM; EEL: electrostatic energy as calculated by the MM force field; EPB: electrostatic contribution to the solvation free energy; NON-POLAR: non-polar solvation energy. TOTAL: total binding free energy from MM/PBSA

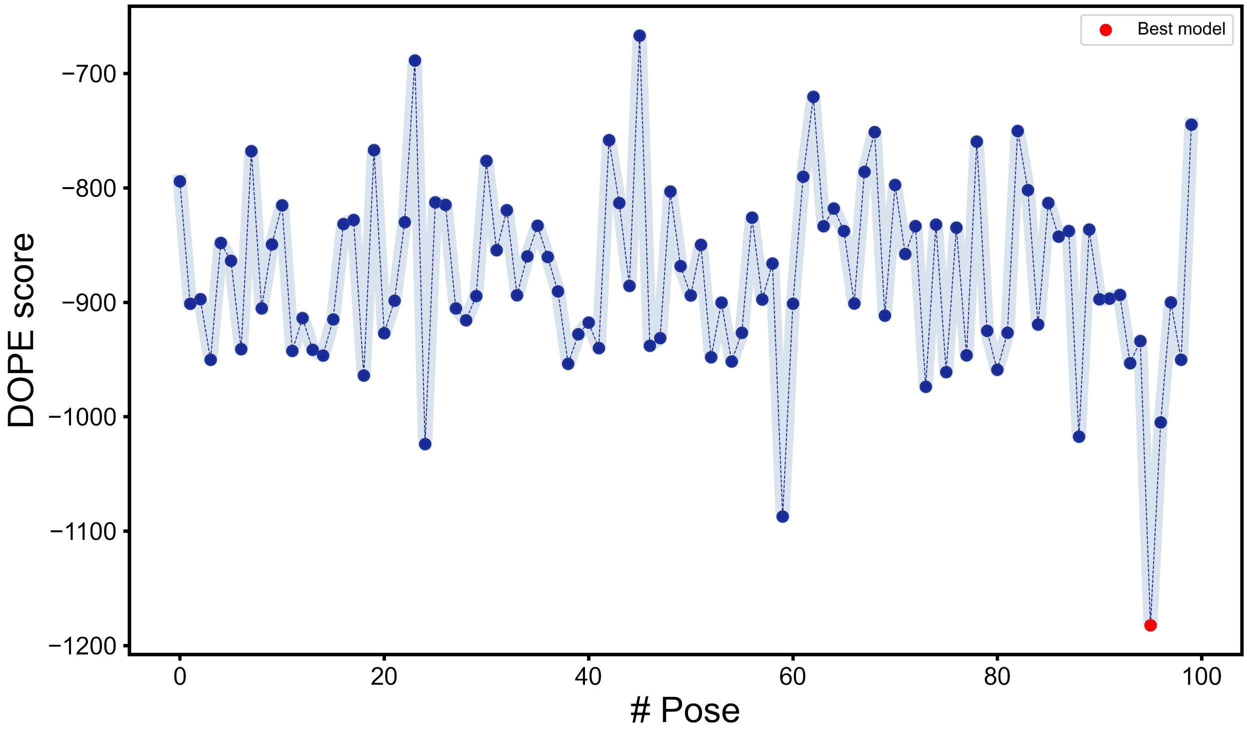

Figure S9 – DOPE Scores of Homology Models. DOPE (Discrete Optimized Protein Energy) scores of the 100 models generated using MODELLER for SIRT1.

A)

```
>Structure:5BTR
---DNLLFGDEIITNG-----------------SDWTPRPRIGPYTFVQQHLMIGTDPRTILKDLLPETIPPPEL
DDMTLWQIVINILSEPPKRKKRKDINTIEDAVKLLQESKKIIVLTGAGVSVSSGIPDFRSRDGIYARLAVDFPDL
PDPQAMFDIEYFRKDPRPFFKFAKEIYPGQFQPSLCHKFIALSDKEGKLLRNYTQNIDTLEQVAGIQRIIQCHGS
FATASCLICKYKVDCEAVRGDIFNQVVPRCPRCPADEPLAIMKPEIVFFGENLPEQFHRAMKYDKDEVDLLIVIG
SSLKVRPVALIPSSIPHEVPQILINREPLPHLHFDVELLGDCDVIINELCHRLGGEYAKLSSNPVKLSEITEQYL
FLPPNRYIFHGAEVY--------*
>Sequence:5BTR missing residues solved
GSRDNLLFGDEIITNGFHSCESDEEDRASHASSSDWTPRPRIGPYTFVQQHLMIGTDPRTILKDLLPETIPPPEL
DDMTLWQIVINILSEPPKRKKRKDINTIEDAVKLLQESKKIIVLTGAGVSVSSGIPDFRSRDGIYARLAVDFPDL
PDPQAMFDIEYFRKDPRPFFKFAKEIYPGQFQPSLCHKFIALSDKEGKLLRNYTQNIDTLEQVAGIQRIIQCHGS
FATASCLICKYKVDCEAVRGDIFNQVVPRCPRCPADEPLAIMKPEIVFFGENLPEQFHRAMKYDKDEVDLLIVIG
SSLKVRPVALIPSSIPHEVPQILINREPLPHLHFDVELLGDCDVIINELCHRLGGEYAKLSSNPVKLSEITEQYL
FLPPNRYIFHGAEVYSDSEDDV*
```

B)

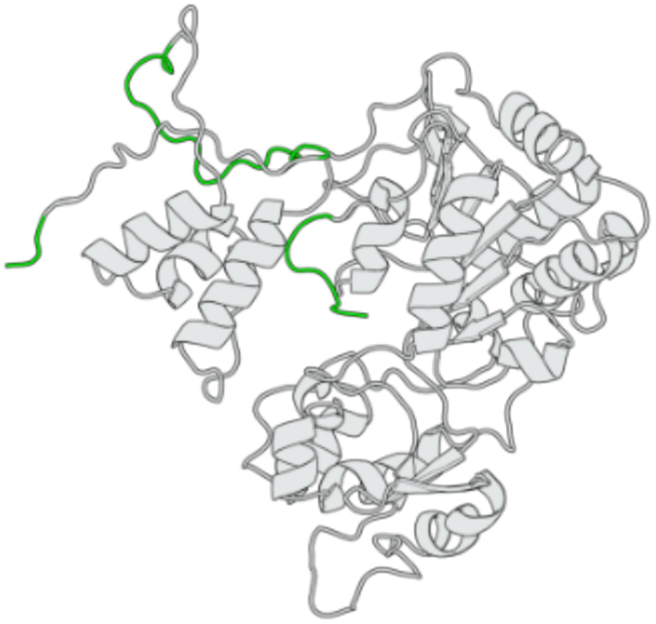

Figure S10 – Sequence Alignment and Homology Model of SIRT1. **A)** Sequence alignment between the full-length SIRT1 amino acid sequence (FASTA) and the crystallographic structure used as a template (PDB ID: 5BTR). Missing regions in the template and reconstructed via homology modeling are shown in green. **B)** 3D structure of the final homology-modeled SIRT1 protein. Residues added during model reconstruction are highlighted in green.

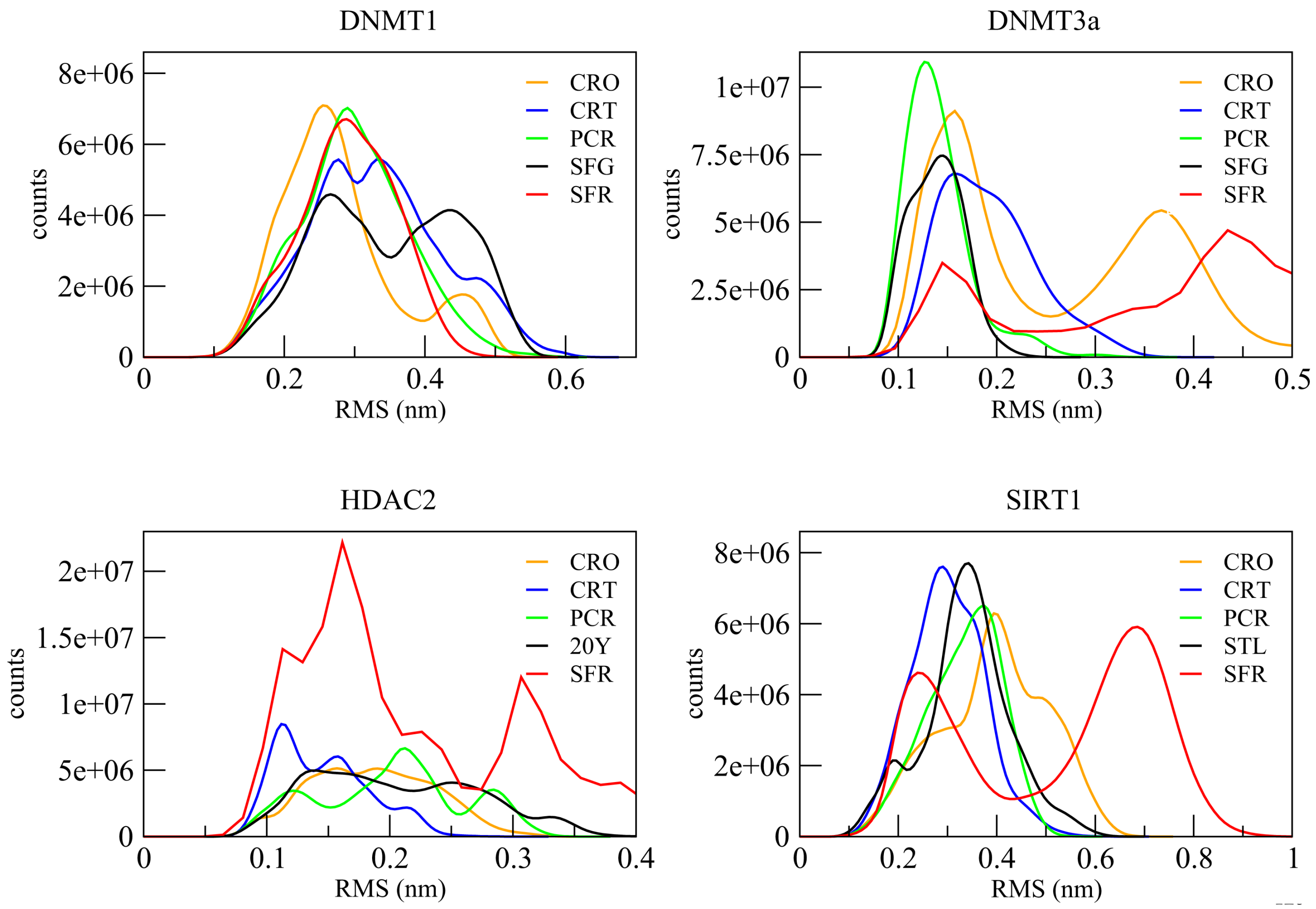

Figure S11 – Distribution of pairwise RMSD values. The RMSD distribution was obtained from the pairwise RMSD matrix calculated over the MD trajectory.

Table S1 – Cluster Analysis. Total number of clusters (tot); the id (#) number and the size (%) of the first three populated clusters for each complex.

Ligand	***DNMT1***			***DNMT3a***			***HDAC2***			***SIRT1***		
	tot	*id*	*size (%)*	*tot*	*id*	*size (%)*	*tot*	*id*	*size (%)*	*tot*	*id*	*size (%)*
SFG/20Y/STL		*#1*	*40.16*		*#1*	*97.60*		*#1*	*50.31*		*#1*	*67.60*
	11	*#2*	*31.47*	*5*	*#2*	*1.57*	*12*	*#2*	*37.13*	*12*	*#2*	*18.17*
		#3	*16.68*		*#3*	*0.80*		*#3*	*6.98*		*#3*	*5.14*
CRO		*#1*	*81.88*		*#1*	*47.10*		*#1*	*53.79*		*#1*	*47.24*
	7	*#2*	*7.45*	*42*	*#2*	*26.14*	*13*	*#2*	*32.76*	*14*	*#2*	*23.39*
		#3	*6.48*		*#3*	*8.62*		*#3*	*5.05*		*#3*	*11.32*
CRT		*#1*	*46.10*		*#1*	*55.07*		*#1*	*88.18*		*#1*	*88.96*
	16	*#2*	*21.10*	*27*	*#2*	*24.96*	*9*	*#2*	*9.49*	*7*	*#2*	*6.02*
		#3	*17.73*		*#3*	*8.49*		*#3*	*1.60*		*#3*	*2.50*
PCR		*#1*	*52.55*		*#1*	*94.18*		*#1*	*50.03*		*#1*	*65.93*
	10	*#2*	*22.61*	*8*	*#2*	*4.24*	*15*	*#2*	*27.94*	*9*	*#2*	*23.53*
		#3	*14.37*		*#3*	*0.70*		*#3*	*8.93*		*#3*	*7.89*
SFR		*#1*	*57.19*		*#1*	*15.59*		*#1*	*57.25*		*#1*	*50.77*
	9	*#2*	*32.61*	*1285*	*#2*	*8.92*	*85*	*#2*	*17.47*	*19*	*#2*	*19.88*
		#3	*5.98*		*#3*	*4.01*		*#3*	*15.76*		*#3*	*11.41*

Table S2 - *In silico* ADME analysis. Evaluation of the ADME properties of saffron bioactive compounds and natural ligands through *in silico* analysis with SwissADME

Ligand	Canonical SMILES	Formula
CRO	OC[C@H]1O[C@@H](OC(=O)/C(=C/C=C/C(=C/C=C/C=C(/C=C/C=C(/C(=O)O)\C)\C)/C)/C)[C@@H]([C@H]([C@@H]1O)O)O	C26H34O9
CRT	C/C(=C\C=C\C=C(\C=C\C=C(\C(=O)O)/C)/C)/C=C/C=C(/C(=O)O)\C	C20H24O4
PCR	OC[C@H]1O[C@@H](O[C@@H]2CC(=C(C(C2)(C)C)C=O)C)[C@@H]([C@H]([C@@H]1O)O)O	C16H26O7
SFR	O=CC1=C(C)C=CCC1(C)C	C10H14O
20Y	CC(=O)Nc1ccc(cc1)C(=O)Nc1cc(ccc1N)c1cccs1	C19H17N3O2S
SFG	N[C@H](C[C@H]1O[C@H]([C@@H]([C@@H]1O)O)n1cnc2c1ncnc2N)CC[C@@H](C(=O)O)N	C15H23N7O5
STL	Oc1ccc(cc1)/C=C/c1cc(O)cc(c1)O	C14H12O3

Ligand	MW	#Heavy atoms	#Aromatic heavy atoms	Fraction Csp3	#Rotatable bonds	#H-bond acceptors	#H-bond donors
CRO	490.54	35	0	0.38	11	9	5
CRT	328.40	24	0	0.20	8	4	2
PCR	330.37	23	0	0.81	4	7	4
SFR	150.22	11	0	0.50	1	1	0
20Y	351,42	25	17	0,05	6	2	3
SFG	381,39	27	9	0,6	7	10	6
STL	228,24	17	12	0	2	3	3

Ligand	MR	TPSA	iLOGP	XLOGP3	WLOGP	MLOGP	Silicos-IT Log P	Consensus Log P
CRO	130.45	153.75	2.13	2.14	1.87	2.10	2.62	2.30
CRT	98.48	74.60	2.13	2.14	4.61	2.10	2.62	2.30
PCR	81.08	116.45	1.77	-0.50	-0.49	-0.88	0.20	0.02
SFR	47.06	17.07	2.13	2.14	2.49	2.10	2.62	2.30
20Y	102,69	112,46	2,21	2,59	3,83	2,22	3,74	2,92
SFG	92,73	208,65	0,67	-4,31	-2,38	-5,13	-3,03	-2,84
STL	67,88	60,69	1,71	3,13	2,76	2,26	2,57	2,48

Ligand	ESOL Log S	ESOL Solubility (mg/ml)	ESOL Solubility (mol/l)	ESOL Class
CRO	-2.05	1.33e+00	8.84e-03	Soluble
CRT	-2.05	1.33e+00	8.84e-03	Soluble
PCR	-1.31	1.62e+01	4.91e-02	Very soluble
SFR	-2.05	1.33e+00	8.84e-03	Soluble
20Y	-3,76	0,0614	0,000175	Soluble
SFG	0,73	2030	5,32	Highly soluble
STL	-3,62	0,0551	0,000241	Soluble

Ligand	Ali Log S	Ali Solubility (mg/ml)	Ali Solubility (mol/l)	Ali Class
**CRO**	-2.13	1.11e+00	7.41e-03	Soluble
**CRT**	-2.13	1.11e+00	7.41e-03	Soluble
**PCR**	-1.48	1.10e+01	3.33e-02	Very soluble
**SFR**	-2.13	1.11e+00	7.41e-03	Soluble
**20Y**	-4,6	0,00882	0,0000251	Moderately soluble
**SFG**	0,54	1320	3,46	Highly soluble
**STL**	-4,07	0,0193	0,0000844	Moderately soluble

Ligand	Silicos-IT LogSw	Silicos-IT Solubility (mg/ml)	Silicos-IT Solubility (mol/l)	Silicos-IT class
**CRO**	-2.13	1.12e+00	7.43e-03	Soluble
**CRT**	-2.13	1.12e+00	7.43e-03	Soluble
**PCR**	-0.18	2.18e+02	6.59e-01	Soluble
**SFR**	-2.13	1.12e+00	7.43e-03	Soluble
**20Y**	-6,69	0,0000719	0,000000205	Poorly soluble
**SFG**	0,34	825	2,16	Soluble
**STL**	-3,29	0,118	0,000516	Soluble

Ligand	GI absorption	BBB permeant	Pgp substrate	CYP1A2 inhibitor	CYP2C19 inhibitor	CYP2C9 inhibitor	CYP2D6 inhibitor	CYP3A4 inhibitor	log Kp (cm/s)
**CRO**	High	Yes	No	No	No	No	No	No	-5.70
**CRT**	High	Yes	No	No	No	No	No	No	-5.70
**PCR**	High	No	Yes	No	No	No	No	No	-8.67
**SFR**	High	Yes	No	No	No	No	No	No	-5.70
**20Y**	High	No	Yes	Yes	Yes	Yes	Yes	Yes	-6,6
**SFG**	Low	No	No	No	No	No	No	No	-11,69
**STL**	High	Yes	No	Yes	No	Yes	No	Yes	-5,47

Ligand	Lipinski #violations	Ghose #violations	Veber #violations	Egan #violations	Muegge #violations	Bioavailability Score
**CRO**	0	1	0	0	2	0.55
**CRT**	0	1	0	0	2	0.55
**PCR**	0	1	0	0	0	0.55
**SFR**	0	1	0	0	2	0.55
**20Y**	0	0	0	0	0	0,55
**SFG**	2	1	1	1	3	0,17
**STL**	0	0	0	0	0	0,55

Ligand	PAINS #alerts	Brenk #alerts	Leadlikeness #violations	Synthetic Accessibility
**CRO**	0	1	1	3.42
**CRT**	0	1	1	3.42
**PCR**	0	1	0	5.29
**SFR**	0	1	1	3.42
**20Y**	0	1	1	2,65
**SFG**	0	0	1	4,74
**STL**	0	1	1	2,02

Table S3 – Ligand-Target Complexes. ID of the ligand-target complexes.

Ligand	***DNMT1***	***DNMT3a***	***HDAC2***	***SIRT1***
SFG/20Y/STL[a]	*DNMT1:SFG*	*DNMT3A:SFG*	*HDAC2:20Y*	*SIRT1:STL*
Crocin	*HDAC2:CRO*	*SIRT1:CRO*	*DNMT1:CRO*	*DNMT3A:CRO*
Crocetin	*HDAC2:CRT*	*SIRT1:CRT*	*DNMT1:CRT*	*DNMT3A:CRT*
Picrocrocin	*HDAC2:PCR*	*SIRT1:PCR*	*DNMT1:PCR*	*DNMT3A:PCR*
Safranal	*HDAC2:SFR*	*SIRT1:SFR*	*DNMT1:SFR*	*DNMT3A:SFR*

NOTES: (a) Abbreviations: SFG: sinefungin inhibitor of DNMT1 and DNMT3a; 20Y: 4-(acetylamino)-N-[2-amino-5-(thiophen-2-yl)phenyl]benzamide inhibitor of HDAC2; STL: resveratrol molecule, activator of SIRT1; CRT: crocetin; CRO, crocin; SFR, safranal; PCR, picrocrocin

Table S4. Docking pose stability analysis

**Ranking predicted affinity docking poses (kcal/mol)**											
**DNMT1**						**DNMT3a**					
	**SFG**	**CRO**	**CRT**	**PCR**	**SFR**		**SFG**	**CRO**	**CRT**	**PCR**	**SFR**
**Pose 1**	-9.3	-8.9	-7.8	-8.2	-6.0	**Pose 1**	-8.1	-9.2	-8.3	-7.2	-5.2
Pose 2	-8.9	-8.8	-7.7	-8.2	-5.7	Pose 2	-7.9	-8.6	-8.1	-7.1	-5.0
Pose 3	-8.7	-8.6	-7.5	-8.1	-5.7	Pose 3	-7.9	-8.5	-8.1	-7.0	-4.9
Pose 4	-8.5	-8.5	-7.5	-7.9	-5.7	Pose 4	-7.9	-8.4	-7.9	-6.9	-4.9
Pose 5	-8.3	-8.5	-7.5	-7.6	-5.6	Pose 5	-7.8	-8.4	-7.9	-6.8	-4.9
Pose 6	-8.2	-8.4	-7.4	-7.6	-5.6	Pose 6	-7.8	-8.3	-7.8	-6.8	-4.9
Pose 7	-8.2	-8.4	-7.4	-7.5	-5.5	Pose 7	-7.7	-8.3	-7.8	-6.8	-4.8
Pose 8	-8.0	-8.4	-7.4	-7.5	-5.5	Pose 8	-7.7	-8.3	-7.5	-6.6	-4.8
Pose 9	-7.9	-8.4	-7.3	-7.4	-5.5	Pose 9	-7.7	-8.3	-7.3	-6.6	-4.8
Pose 10	-7.9	-8.2	-7.3	-7.3	-5.7	Pose 10	-7.5	-8.2	-7.2	-6.4	-4.8
**HDAC2**						SIRT1					
	**20Y**	**CRO**	**CRT**	**PCR**	**SFR**		**SFR**	**CRO**	**CRT**	**PCR**	**SFR**
**Pose 1**	-8.4	-7.3	-7.5	-6.1	-5.6	**Pose 1**	-8.0	-8.5	-8.1	-7.3	-5.2
Pose 2	-7.7	-7.0	-7.2	-5.9	-5.1	Pose 2	-7.9	-8.3	-7.9	-7.2	-5.1
Pose 3	-7.4	-7.0	-7.1	-5.7	-5.0	Pose 3	-7.6	-8.2	-7.9	-7.0	-5.1
Pose 4	-7.1	-6.8	-6.9	-5.4	-4.9	Pose 4	-7.6	-8.2	-7.8	-7.0	-5.1
Pose 5	-6.8	-6.7	-6.7	-5.3	-4.9	Pose 5	-7.2	-8.1	-7.8	-6.9	-5.0
Pose 6	-6.8	-6.7	-6.7	-5.2	-4.9	Pose 6	-7.2	-8.1	-7.7	-6.9	-5.0
Pose 7	-6.7	-6.7	-6.6	-5.2	-4.9	Pose 7	-7.1	-8.1	-7.6	-6.9	-4.9
Pose 8	-6.7	-6.7	-6.6	-5.0	-4.8	Pose 8	-7.1	-8.1	-7.5	-6.8	-4.9
Pose 9	-6.6	-6.6	-6.6	-5.0	-4.8	Pose 9	-7.1	-8.0	-7.4	-6.8	-4.9
Pose 10	-6.6	-6.4	-6.6	-4.9	-4.8	Pose 10	-7.1	-8.0	-7.3	-6.8	-4.9

Table S5 – Equilibration Procedure

	t	Δt	T	F_c	τ_t	τ_p
	[ps]	[fs]	[K]	[KJ/mol .nm^2]	[ps]	[ps]
NVT step1	2	1	50	100000	0.01[(a)] 0.01[(b)]	-
NVT step2	4	1	100	100000	0.01 0.01	-
NVT step3	6	1	150	100000	0.03 0.01	-
NVT step4	8	1	200	100000	0.03 0.01	-
NVT step5	10	1	250	100000	0.03 0.01	-
NVT step6	12	1	300	100000	0.03 0.01	-
NVT step7	100	2	300	70000	0.03 0.01	-
NVT step8	150	2	300	50000	0.03 0.01	-
NVT step9	200	2	300	30000	0.03 0.01	-
NVT step10	250	2	300	10000	0.01 0.01	-
NVT step11	300	2	300	3000	0.01 0.01	-
NPT	500	2	300	-	0.01 0.01	2.0

NOTES: t, simulation time; Δt, timestep; T, temperature; F_c, positional restraint force; τ_t, temperature coupling constant for the solute[(a)] and solvent[(b)], τ_p, pressure bath coupling
